# Are dopamine agonists still the first-choice treatment for prolactinoma in the era of endoscopy? A systematic review and meta-analysis

**DOI:** 10.1186/s41016-022-00277-1

**Published:** 2022-04-08

**Authors:** Xiangming Cai, Junhao Zhu, Jin Yang, Chao Tang, Zixiang Cong, Chiyuan Ma

**Affiliations:** 1 School of Medicine, Southeast University, Nanjing, China; 2 School of Medicine, Nanjing Medical University, Nanjing, China; 3 Department of Neurosurgery, Jinling Hospital, Nanjing, China; 4 School of Medicine, Nanjing University, Nanjing, China

**Keywords:** Prolactinoma, Dopamine agonists, Bromocriptine, Cabergoline, Microscopic surgery, Endoscopic surgery

## Abstract

**Background:**

For prolactinoma patients, dopamine agonists (DAs) are indicated as the first-line treatment and surgery is an adjunctive choice. However, with the development of surgical technique and equipment, the effect of surgery has improved. The aim of this study was to assess the efficacy and safety of surgery versus DAs in patients with different types of prolactinomas.

**Methods:**

A systematic search of literature using Web of Science, PubMed, Cochrane Library, and Clinical Trial databases was conducted until July 12, 2019. Prolactinoma patients treated with DAs (bromocriptine or cabergoline) or surgery (microscopic or endoscopic surgery) were included. Outcomes included the biochemical cure rate, recurrence rate, prolactin level, improvement rates of symptoms, and incidence rates of complications. A random-effects model was used to pool the extracted data. Qualitative comparisons were conducted instead of quantitative comparison.

**Results:**

DAs were better than surgery in terms of the biochemical cure rate (0.78 versus 0.66), but surgery had a much lower recurrence rate (0.19 versus 0.57). Full advantages were not demonstrated in improvement rates of symptoms and incidence rates of complications with both treatment options. In microprolactinoma patients, the biochemical cure rate of endoscopic surgery was equal to the average cure rate of DAs (0.86 versus 0.86) and it surpassed the biochemical cure rate of bromocriptine (0.86 versus 0.76). In macroprolactinoma patients, endoscopic surgery was slightly higher than bromocriptine (0.66 versus 0.64) in terms of the biochemical cure rate.

**Conclusion:**

For patients with clear indications or contraindications for surgery, choosing surgery or DAs accordingly is unequivocal. However, for patients with clinical equipoise, such as surgery, especially endoscopic surgery, in microprolactinoma and macroprolactinoma patients, we suggest that neurosurgeons and endocrinologists conduct high-quality clinical trials to address the clinical equipoise quantitatively.

**Supplementary Information:**

The online version contains supplementary material available at 10.1186/s41016-022-00277-1.

## Background

Prolactinomas are the most common type of hormone-secreting pituitary tumors and they represent 40% of all pituitary tumors [1]. Dopamine agonists (DAs), including bromocriptine and cabergoline, are recommended as the first-line treatment for most prolactinomas. Surgery is only an adjunctive choice when resistance or intolerance to DAs occurs or severe complications, such as pituitary apoplexy or cerebrospinal fluid leak, develop [2].

However, with the development of surgical technique and equipment, especially endoscopic surgery, it is time to reassess the relationship between DAs and surgery. Only few retrospective studies [3–8] have compared the efficacy and safety between surgery and DAs in some specific subgroups of prolactinoma patients. And few meta-analyses discussed the difference among treatments for prolactinoma in some outcomes, mostly remission rates and recurrence rates [9–11]. As far as we know, no meta-analysis discussed comprehensive efficacy (remission and symptom relief) and safety (relapse and complications) for various treatments of a full spectrum of prolactinoma patients. Because of the lack of a large sample-sized study comparing these two methods in all prolactinoma patients, we conducted this meta-analysis to compare the efficacy and safety of surgery versus DAs in all prolactinoma patients with a focus on the following outcomes: biochemical cure rate, recurrence rate, symptom improvement rates, and incidence rates of complications.

## Methods

This study was conducted in accordance with PRISMA (Preferred Reporting Items for Systematic Review and Meta-Analysis) [12].

### Literature research

Web of Science, PubMed, Cochrane Library, and Clinical Trial databases were independently searched until September 3, 2019, by Cai and Zhu. Search strategy combined MESH terms including “Prolactinoma,” “Dopamine Agonists,” “Microscopy,” and “Endoscopy” with free-text words including “Microprolactinoma,” “Macroprolactinoma,” “Giant prolactinoma,” “Bromocriptine,” “Cabergoline,” and “Surgery” (Supplementary file 1). Studies were restricted to the English language in this research.

### Inclusion criteria

The eligibility criteria consisted of the following items: (1) only studies that included patients who had been diagnosed with prolactinoma. Prolactinomas are classified by the size of the tumor as microprolactinoma (< 10 mm), macroprolactinoma (≥ 10 mm), and giant prolactinoma (> 40 mm) [13]; (2) required treatments included surgery (microscopic surgery or endoscopic surgery) or DAs (bromocriptine or cabergoline). Patients in the DAs group only received DAs, but patients in the surgery group may have received DAs before surgery; (3) included studies reported the data of at least one available outcome that was assessed in this study.

### Exclusion criteria

We excluded the following studies: (1) papers that assessed other pituitary tumors; (2) studies that utilized other DAs, gamma knife surgery, or radiation therapy; (3) studies that included less than 10 patients.

### Extraction of data

Following data were extracted from each paper: author, year of publication, subtype of prolactinoma, intervention, size of sample, gender proportion, mean age, and mean follow-up duration. We also assessed the biochemical cure rate, recurrence rate, and the following variables before and after treatment: prolactin level, visual impairment, headache, menstrual disturbance, galactorrhoea, adrenocorticotropic hormone (ACTH) insufficiency, thyroid-stimulating hormone (TSH) deficiency, hypopituitarism (one or more deficiencies), and diabetes insipidus. Recurrence was defined as the observation of hyperprolactinemia after a period of normalization after surgery and withdrawal of DAs. The assessment of hormonal deficiencies was performed by calculating the presence of hormonal deficiencies after treatment. The extraction of data was independently carried out by Cai and Zhu.

### Quality assessment

The same two reviewers (Cai and Zhu) assessed risk of bias for included studies independently. ROB 2 Cochrane risk of bias tool was used for the randomized controlled trials (RCTs) and ROBINS-I tool for non-randomized controlled trials (non-RCTs) [14, 15]. As no available text-book quality guidelines for case-series studies, we used a tool developed by Moga et al. to assess case-series studies [16]. No cutoff scores were provided within this tool, so we gave one point to each “yes” answer and zero to each “no” and “unclear” answer.

### Statistical analysis

To conduct a meta-analysis of single rates, STATA Version 12.0 and MetaAnalyst Beta 3.13 were applied separately for assessing the biochemical cure rate, recurrence rate, and other parameters. A RE (random-effects) model using Mantel-Haenszel heterogeneity method was also used in these two programs. RevMan Version 5.0 was used to evaluate the pooled mean difference between pre- and post-treatment prolactin levels using the RE model. With this procedure, *I*-squared values were calculated to assess the heterogeneity of pooled results. Subgroup analysis and meta-regression analysis of mean age, gender, publication year, subtypes of prolactinoma, subtypes of surgery, and drug species were conducted to discover the sources of heterogeneity. A funnel plot was used to evaluate the publication bias. As the indications for surgery and DAs were significantly different from each other, we only conducted qualitative comparison instead of formal quantitative comparison in the meta-analysis.

## Results

### Included studies

**Fig. 1:**
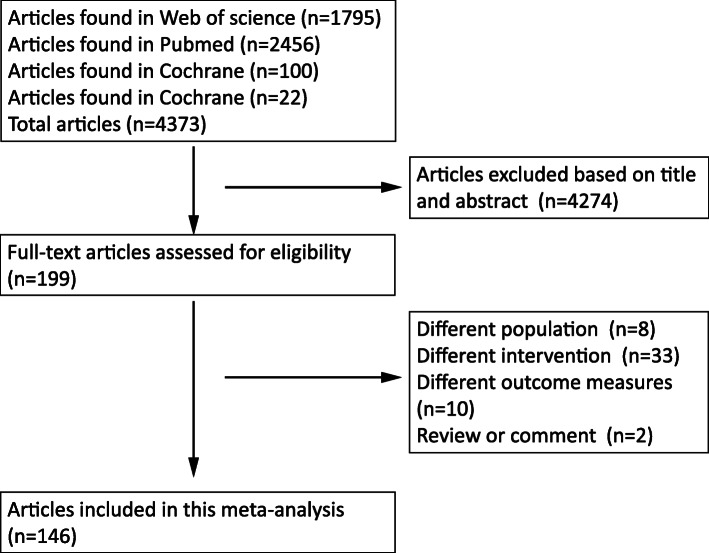
Literature research result

Based on our search strategy, 4373 papers were identified in the databases. From these 4373 papers, 4174 papers were excluded after screening the titles and abstracts (Fig. 1). The remaining 199 full-text articles were assessed for eligibility. During this process, 53 articles were excluded because of differences in the population, interventions, outcomes, or type of articles compared with inclusion criteria.

Finally, a total of 146 articles were included in this meta-analysis. Further, 82 of these 146 articles provided data for the DAs group [3–8, 13, 17–91] and 72 articles provided data for the surgery group [3–8, 13, 68, 92–155]. Details of these 146 studies are presented in Table 1 and Supplementary Tables 1 and 2 separately. The meta-analysis included 9007 patients with no restriction on age and gender. Most studies reported the biochemical cure rates after treatment, but the recurrence rates were provided only in most studies on surgery and few studies on DAs focusing on withdrawal of medicine.

**Table 1:** Basic characteristics of the included studies

Study name	I/A/G^a^	Intervention^**b**^	No.	Male/female	Meanage/y	Biochemical cure rate^**c**^	Recurrent rate^**d**^	Duration 1	Duration 2	Duration 3	Study type
**Adam 2013**	mixed_p	endoscopic_s	17	NA	NA	8/17	NA	40			Case-series
**Akira 2006**	mixed_p	mixed_s	13	3/10	NA	NA	NA	NA			Case-series
**Albert 1992**	0/29/0	BRC	29	14/15	NA	NA	NA	NA	NA	NA	Non-RCT
**Alessandro 2013**	mixed_p	CAB	43	8/35	33.65	24/43	NA	NA	12	NA	Case-series
**Alexander 2018**	60/0/0	endoscopic_s	60	10/50	33.5	40/60	NA	37			Non-RCT
**Amir 2007**	12/13/0	endoscopic_s	25	NA	NA	21/25	NA	19			Case-series
**Amit 2015**	0/71/0	CAB	71	71/0	44.7	51/71	NA	80.3	NA	NA	Case-series
**Andreja 2012**	39/22/0	endoscopic_s	61	NA	NA	54/61	NA	NA			Case-series
**Annamaria1 2004**	mixed_p	CAB	20	20/0	34	20/20	NA	NA	NA	NA	Non-RCT
**Annamaria2 2004**	10/41/0	CAB	51	51/0	32.9	39/51	NA	24			Non-RCT
**Annamaria 2007**	115/79/0	CAB	194	NA	NA	NA	81/194	68.6	42.6	45.8	Case-series
**Annamaria 1997**	8/19/0	mixed_DA	27	NA	NA	23/27	NA	NA	NA	NA	Case-series
**Annamaria 2000**	0/45/0	mixed_DA	45	17/45	NA	40/45	NA	NA	NA	NA	Non-RCT
**Antonell 2001**	44/28/0	mixed_DA	188	NA	NA	138/188	NA	8.3	NA	NA	Non-RCT
**Antonio 2007**	mixed_p	mixed_s	65	20/45	36	42/65	6/42	56			Case-series
**Arafah 1986**	mixed_p	microscopic_s	120	0/120	27.9	96/120	NA	NA			Case-series
**Archer 1982**	17/0/0	BRC	17	0/17	NA	16/17	NA	24	24	NA	Case-series
**Arijit 2005**	0/15/14	BRC	29	29/0	31.9	NA	NA	NA	NA	NA	Case-series
**Arimantas 2012**	32/0/0	microscopic_s	32	0/32	31	19/32	NA	50.4			Case-series
**Arturo 1979**	mixed_p	BRC	14	0/14	29.71	10/14	NA	NA	NA	NA	Case-series
**Asano 2001**	mixed_p	mixed_t	13	NA	37.3	NA	NA	NA			Non-RCT
**Ashu 2013**	0/38/0	CAB	38	21/17	34.2	33/38	NA	16.1	NA	NA	RCT
**Ashu 2012**	0/38/0	CAB	38	NA	NA	30/38	NA	6	NA	NA	RCT
**Barbara 2017**	mixed_p	BRC	28	0/28	26	13/28	NA	NA	NA	NA	Case-series
**Barbosa 2014**	mixed_p	mixed_DA	21	NA	NA	17/21	NA	NA	6	NA	Non-RCT
**Berezin 1995**	mixed_p	mixed_t	75	75/0	NA	36/52	NA	NA	NA	NA	Case-series
**Bevan 1987**	mixed_p	mixed_s	67	19/48	32.4	34/67	NA	NA			Case-series
**Bhansali 2010**	0/15/0	CAB	15	NA	31.7	14/15	NA	NA	NA	NA	Case-series
**Biswas 2005**	89/0/0	mixed_DA	89	NA	NA	NA	57/89	37.2	37.2	21.6	Non-RCT
**Cannavo 1999**	26/11/0	CAB	37	5/32	NA	34/37	NA	NA	24	NA	Case-series
**Carlo 1992**	mixed_p	CAB	127	3/124	NA	114/127	NA	NA	14	NA	Case-series
**Catarina 2018**	0/67/0	mixed_DA	67	34/33	43	58/67	NA	NA	NA	NA	Case-series
**Charpentier 1985**	mixed_p	mixed_s	212	NA	NA	96/212	12/70	52.8			Case-series
**Christine 2016**	0/57/0	mixed_DA	57	30/27	37.5	NA	NA	NA	NA	NA	Non-RCT
**Cintia 2011**	mixed_p	mixed_DA	22	NA	NA	17/22	NA	NA	6	NA	Non-RCT
**Coculescu 1983**	mixed_p	BRC	22	NA	NA	19/22	NA	NA	10.1	NA	Case-series
**Corsello 2003**	0/0/10	CAB	10	NA	NA	5/10	NA	NA	38.9	NA	Case-series
**Der-Yang 2002**	mixed_p	mixed_s	44	1/43	46	32/44	NA	NA			RCT
**Diane 2017**	27/50/0	mixed_s	77	NA	NA	40/77	8/36	12			Case-series
**Dogan 2015**	42/0/0	CAB	42	NA	NA	NA	34/42	12	NA	NA	Non-RCT
**Elise 1984**	42/23/0	mixed_s	65	NA	NA	46/65	6/46	50			Case-series
**Emir 2018**	mixed_p	mixed_DA	25	18/7	39.96	NA	NA	NA	NA	NA	Non-RCT
**Enrica 1989**	mixed_p	mixed_s	22	1/21	NA	NA	NA	NA			Case-series
**Erika1 2007**	mixed_p	mixed_DA	31	0/31	33.0	NA	NA	NA	NA	NA	Non-RCT
**Erika2 2007**	mixed_p	mixed_DA	45	0/45	34.5	NA	NA	NA	NA	NA	Non-RCT
**Esposito 2004**	mixed_p	mixed_s	42	14/26	33.2	25/42	5/21	31			Case-series
**Essais 2002**	0/29/0	BRC	29	10/19	NA	27/29	NA	NA	NA	NA	Case-series
**Etienne 1996**	mixed_p	mixed_DA	10	2/8	NA	8/9	NA	NA	NA	NA	Non-RCT
**Etienne 2009**	0/122/0	CAB	122	50/72	NA	115/122	NA	NA	NA	NA	Case-series
**Etual 2016**	0/152/47	mixed_DA	199	114/85	40.9	145/199	NA	NA	NA	NA	Non-RCT
**Eun-Hee 2009**	0/10/0	CAB	10	10/0	37	6/10	NA	NA	19	NA	Case-series
**Fadi 1996**	mixed_p	mixed_s	64	NA	NA	59/64	25/59	147.6			Case-series
**Ferrari 1997**	0/85/0	CAB	85	NA	NA	52/85	NA	NA	NA	NA	Case-series
**Frederick 2018**	mixed_p	endoscopic_s	79	22/57	35.8	65/79	NA	NA			Non-RCT
**Fritz 1985**	13/11/0	mixed_s	24	0/24	29.7	NA	14/24	NA			Case-series
**Giorgio 2006**	28/38/0	endoscopic_s	66	NA	NA	50/66	NA	NA			Case-series
**Giulio 1989**	mixed_p	mixed_s	119	0/119	NA	73/119	5/40	NA			Case-series
**Hae-Dong 2001**	mixed_p	endoscopic_s	35	NA	NA	24/35	NA	NA			Case-series
**Hae-Dong 1997**	mixed_p	endoscopic_s	15	2/13	32.2	10/15	NA	NA			Case-series
**Hamilton 2005**	mixed_p	mixed_s	79	NA	NA	34/79	NA	NA			Non-RCT
**Hancock 1980**	mixed_p	BRC	36	NA	NA	28/36	NA	NA	NA	NA	Case-series
**Helen 1999**	32/0/0	mixed_s	32	0/32	NA	25/32	1/25	70			Case-series
**Hidemitsu 2001**	mixed_p	microscopic_s	13	NA	NA	NA	NA	NA			Case-series
**Hidetoshi 2013**	mixed_p	mixed_s	138	NA	NA	105/138	5/81	144			Case-series
**Hildebrandt 1989**	0/10/0	BRC	10	NA	NA	3/10	NA	NA	1	NA	Case-series
**Hildebrandt 1992**	mixed_p	mixed_DA	14	NA	NA	10/14	NA	NA	NA	NA	Non-RCT
**Hofstetter 2011**	32/53/0	endoscopic_s	85	NA	NA	51/85	NA	NA			Case-series
**Huda 2010**	40/0/0	mixed_DA	40	1/39	NA	NA	31/40	58	108	58	Case-series
**Ilan 2007**	0/0/10	CAB	10	10/0	38.2	9/10	NA	NA	NA	NA	Case-series
**Ilan 2016**	0/0/18	mixed_DA	18	16/2	36.3	11/18	NA	NA	NA	NA	Case-series
**Ilan 2019**	mixed_p	mixed_DA	28	28/0	71.3	24/27	NA	NA	NA	NA	Case-series
**Ivan 2015**	40/38/0	mixed_t	78	23/55	39.8	44/78	NA	NA	25	NA	Non-RCT
**Jackson 2010**	7/34/0	endoscopic_s	41	NA	NA	34/41	3/35	NA			Case-series
**Jae 2009**	mixed_p	mixed_t	117	31/86	35.1	103/117	NA	NA	NA	NA	Case-series
**Johanna 1991**	0/12/0	BRC	12	8/4	42.2	NA	11/12	12	58.8	4.3	Case-series
**Johanna 1990**	0/19/0	BRC	19	12/7	NA	16/19	NA	40.8	40.8	NA	Case-series
**Jonathan 1992**	mixed_p	mixed_s	82	7/75	30.5	65/82	5/65	51.7			Case-series
**Katarina 2011**	mixed_p	mixed_DA	14	6/8	39.7	14/14	NA	NA	6	NA	Case-series
**Kharlip 2009**	mixed_p	CAB	46	NA	NA	NA	25/46	NA	NA	3	Case-series
**Kiyoshi 1984**	mixed_p	mixed_s	12	NA	NA	NA	NA	NA			Case-series
**Kreutzer 2008**	mixed_p	mixed_s	212	133/79	36	102/212	17/91	NA			Non-RCT
**Kristof 2002**	mixed_p	mixed_s	37	16/21	31	10/37	2/10	44.4			Case-series
**Kyung 2013**	mixed_p	BRC	23	17/6	48	16/23	NA	NA	30	NA	Case-series
**Liang 2018**	0/0/42	mixed_t	42	NA	NA	21/42	NA	NA	NA	NA	Non-RCT
**Lukas 2017**	mixed_p	mixed_t	107	0/107	34	65/107	NA	NA	NA	NA	Non-RCT
**Marco 2002**	mixed_p	mixed_s	120	27/93	29.7	77/120	13/77	50.2			Case-series
**Margarida 2017**	mixed_p	mixed_DA	50	5/45	35.1	NA	14/50	NA	119.3	NA	Non-RCT
**Maria 2015**	mixed_p	mixed_DA	29	NA	NA	29/29	NA	NA	NA	NA	Case-series
**María Martín 2013**	47/0/0	mixed_DA	47	NA	30	39/47	NA	NA	NA	NA	Case-series
**Mario 2017**	24/0/0	mixed_s	24	5/19	34.8	8/24	1/8	NA			Non-RCT
**Masami 2010**	mixed_p	CAB	85	NA	NA	85/85	NA	NA	NA	NA	Case-series
**Mia-Maiken 2013**	mixed_p	mixed_DA	12	5/7	39.7	8/12	NA	NA	NA	NA	Case-series
**Michael 2009**	mixed_p	mixed_s	176	20/156	31	NA	NA	NA			Non-RCT
**Miguel 1982**	mixed_p	microscopic_s	100	NA	NA	68/100	5/68	NA			Case-series
**Moon 2011**	mixed_p	BRC	36	25/11	NA	29/36	NA	NA	NA	NA	Case-series
**Muratori 1997**	26/0/0	CAB	26	0/26	NA	25/26	13/19	12	12	NA	Case-series
**Muriel 2011**	24/10/0	microscopic_s	34	4/30	NA	32/34	2/32	33.5			Case-series
**Mussa 2015**	0/0/16	CAB	16	10/6	34.9	6/16	NA	NA	NA	NA	Case-series
**Myoung 2017**	30/59/0	mixed_DA	89	27/62	33.7	NA	51/89	25.8	28.9	NA	Case-series
**Na 2018**	31/32/0	mixed_s	63	NA	57	48/63	3/48	53			Case-series
**Naguib 1986**	mixed_p	mixed_t	190	0/190	28.6	NA	NA	NA	28.8	NA	Non-RCT
**Nazir 2015**	mixed_p	CAB	19	1/18	27.3	18/19	NA	NA	NA	NA	Non-RCT
**Niki 2013**	0/12/0	CAB	12	11/1	40.5	11/12	NA	NA	NA	NA	Case-series
**Nissim 1982**	0/7/0	BRC	7	NA	NA	4/7	NA	NA	NA	NA	Case-series
**Oksana 2018**	0/0/68	mixed_t	68	60/8	41.5	35/68	NA	NA	104.7	NA	Case-series
**Oluwaseun 2019**	mixed_p	mixed_DA	69	NA	NA	29/69	NA	6	NA	NA	Case-series
**Omar 1983**	28/16/0	mixed_s	44	0/44	26.8	29/44	16/29	41.5			Case-series
**Paepegaey 2017**	0/260/0	CAB	260	135/125	36.2	157/260	14/35	NA	NA	NA	Case-series
**Paluzzi 2013**	11/42/0	endoscopic_s	53	NA	NA	42/53	NA	NA			Case-series
**Panagiotis 2011**	mixed_p	mixed_DA	79	17/62	35.3	NA	11/26	49	79	NA	Case-series
**Paul 1983**	mixed_p	mixed_s	40	0/40	NA	25/40	9/25	23			Case-series
**Pelkonen 1981**	mixed_p	mixed_s	60	15/45	NA	NA	NA	NA			Case-series
**Pietro 2005**	mixed_p	mixed_s	151	NA	NA	93/151	NA	NA			Case-series
**Raverot 2010**	mixed_p	mixed_s	94	32/62	37.8	60/94	19/60	138			Case-series
**Renata 2013**	mixed_p	CAB	61	13/48	34.4	57/61	NA	60	60	NA	Case-series
**Renata 2015**	mixed_p	CAB	32	32/0	42	31/32	NA	24	24	NA	Non-RCT
**Ronald 1982**	22/14/0	mixed_s	36	NA	NA	NA	1/35	NA			Case-series
**Rudolf 1985**	27/0/0	microscopic_s	27	NA	NA	19/27	NA	NA			Case-series
**Safak 2016**	0/113/0	endoscopic_s	113	NA	NA	51/113	NA	36			Case-series
**Safak 2016**	19/0/10	endoscopic_s	29	NA	NA	15/29	NA	36			
**Sandhya 2018**	mixed_p	mixed_DA	28	0/28	NA	16/18	5/16	12	216	36	Case-series
**Sandhya 2017**	mixed_p	mixed_DA	16	0/16	NA	15/16	NA	NA	NA	NA	Case-series
**Schlechte 1985**	mixed_p	microscopic_s	68	0/68	NA	37/68	12/37	60.00			Case-series
**Sema 2016**	mixed_p	mixed_DA	67	17/50	NA	NA	31/67	108.8	76.9	16.1	Non-RCT
**Sema 2018**	mixed_p	mixed_DA	308	NA	71	NA	NA	NA	NA	NA	Non-RCT
**Shigetoshi 2009**	17/12/0	endoscopic_s	29	NA	NA	21/29	NA	NA			Case-series
**Shrikrishna 2009**	mixed_p	mixed_DA	39	9/30	NA	14/39	NA	NA	NA	NA	Case-series
**Shrikrishna 2010**	0/0/10	CAB	10	5/5	36.1	8/10	NA	NA	NA	NA	Case-series
**Steven 1996**	11/23/0	mixed_s	34	8/26	23.3	9/34	NA	NA			Case-series
**Taizo 1991**	mixed_p	mixed_s	35	0/35	NA	22/35	NA	NA			Case-series
**Takakazu 2002**	mixed_p	mixed_s	32	12/20	32	14/32	NA	NA			Case-series
**Tevfik 2001**	mixed_p	mixed_DA	34	4/30	33.1	24/34	NA	NA	NA	NA	RCT
**Thomas 2011**	45/15/0	mixed_DA	60	NA	NA	NA	43/60	65	59	6	Case-series
**Thomson 1985**	mixed_p	microscopic_s	77	NA	NA	53/77	NA	NA			Case-series
**Timothy 2015**	mixed_p	endoscopic_s	66	22/44	36.7	45/66	NA	12			Case-series
**Vanessa 2012**	mixed_p	mixed_s	63	18/45	31	29/63	10/29	36			Case-series
**Verena 2017**	mixed_p	CAB	53	31/22	40	NA	NA	NA	9	NA	Case-series
**Wang 1987**	mixed_p	BRC	24	NA	NA	NA	19/24	40.8	58.8	NA	Case-series
**Wang 2015**	132/176/0	endoscopic_s	308	NA	NA	261/308	NA	NA			Case-series
**Winnie 2018**	mixed_p	mixed_s	31	31/0	40.8	NA	NA	41.9			Case-series
**Wolfsberger 2003**	0/11/0	mixed_s	11	11/0	41	8/11	NA	84			Case-series
**Xin 2011**	mixed_p	mixed_s	87	87/0	38	46/87	9/45	45			Case-series
**Yan 2015**	mixed_p	mixed_s	99	NA	NA	71/99	NA	NA			Case-series
**Yang 2015**	mixed_p	mixed_s	9	5/4	NA	NA	NA	NA			Case-series
**Yan-Long 2018**	mixed_p	endoscopic_s	52	14/38	37.69	40/52	6/40	13.5			Case-series
**Yi 2018**	mixed_p	mixed_s	36	11/25	NA	34/36	NA	NA			Case-series
**Yi-Jun 2017**	mixed_p	microscopic_s	184	184/0	36.3	57/187	NA	NA			Case-series
**Youichi 1986**	mixed_p	microscopic_s	98	16/82	31	45/98	NA	NA			Case-series
**Youngki 2014**	0/44/0	mixed_DA	44	28/16	36.8	34/44	NA	NA	NA	NA	Case-series

^a^I/A/G: numbers of patients with microprolactinoma/macroprolactinoma/giant prolactinoma; mixed_p: mixed_prolactinoma, data of this part is inseparable, which includes patients with macroprolactinoma, microprolactinoma, and giant prolactinoma; ^b^mixed_t: mixed treatment,treatments within this study include DAs and surgery and data of each treatment is available; mixed_s: mixed_surgery, data include patients with microscopic surgery and endoscopic surgery; microscopic_s: microscopic_surgery; endoscopic_s: endoscopic_surgery; DAs: dopamine agonists; BRC: bromocriptine; CAB: cabergoline; ^c^ cured/treated; ^d^ replased/cured; ^e^ mean follow up duration months; *NA* not applicable, because the data was not provided by included studies. Duration 1: follow up duration (month); Duration 2: DAs treatment duration (month), only for studies with DAs; Duration 3: follow-up duration after DAs withdrawal (month), only for studies with DAs; No.: sample size of included study

Quality assessments showed some concern for most RCTs because of their unclear description about random process and prespecified analysis plan. The assessments also found 18.8% (6/32) high, 21.9% (7/32) moderate, and 59.4% (19/32) low overall bias for non-RCTs, and the main bias was confounding and excluding patients due to missing data. The average score for case series studies was 11.9 [4–16], and the main bias came from study design (Q2–4) and unclear description of statistical analysis (Q14). The summary of risk of bias within studies was provided in Supplementary Fig. 1 and Supplementary Tables 3, 4 and 5.

### Biochemical cure rate

**Fig. 2:**
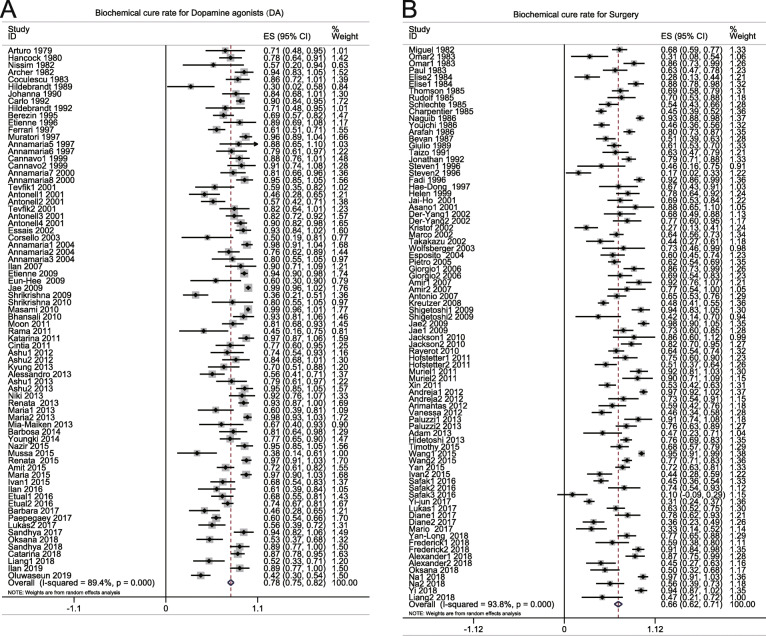
Forest plot for biochemical cure rate in prolactinoma patients treated with DAs (**a**) and patients treated with surgery (**b**)

A total of 81 studies [4–8, 13, 68, 84, 92–97, 99–112, 114, 118, 120–123, 125, 127–133, 135–137, 139, 141–156] comprising 4397 patients who received surgery and 74 studies [3–6, 8, 13, 17–21, 25, 26, 28–36, 38, 42–46, 48–51, 54–58, 60, 61, 65–73, 76, 79–81, 85–87, 89, 91] comprising 2659 patients who used DAs were included in this part of the research. The pooled prolactin normalization rates were 0.66 (0.62, 0.71) (*I*^2^ = 93.8%, *p* = 0.000) in the surgery group and 0.78 (0.75, 0.82) (*I*^2^ = 89.4%, *p* = 0.000) in the DAs group, respectively (Fig. 2). Because of high heterogeneity, subgroup analysis and meta-regression analysis were conducted to detect the source of high heterogeneity. In the surgery group, although no significant decrease in heterogeneity was found in the subgroup analysis (Supplementary Fig. 2), meta-regression analysis detected that gender (*p* = 0.019) and macroprolactinoma (*p* = 0.001) were statistically significant factors causing heterogeneity. In the subgroup analysis, macroprolactinoma patients showed a lower biochemical cure rate (0.57 versus 0.66) compared with total surgery-treated patients, but in macroprolactinoma patients, the biochemical cure rate was higher (0.79 versus 0.66) than total surgery-treated patients (Supplementary Fig. 2). And regression analysis identified that female patients showed a positive trend in the rates compared with male patients. Because the surgery group included patients with or without DAs treatment history, we conducted subgroup analysis based on DAs treatment history to explore the normalization rate of surgery treated population without DAs treatment history. Results showed similar normalization rates in without DAs treatment history subgroup (0.69 (0.44,0.94); *I*^2^ = 94.5%, *p* = 0.000) with that in the whole surgery treated population (Supplementary Fig. 8). In the DAs group, subgroup analysis was carried out based on decades, subtypes of prolactinoma, and drug species (Supplementary Fig. 2), and the giant prolactinoma (*I*^2^ = 62.3%, *p* = 0.010) subgroup showed a decrease in important heterogeneity (Table 2). Meta-regression analysis of the DAs group also showed that giant prolactinoma (*p* = 0.029) and bromocriptine (*p* = 0.024) were important sources of heterogeneity (Table 4), and their rates were lower than the rates in all patients (0.62 versus 0.78; 0.70 versus 0.78). The funnel plot for the surgery group (Supplementary Fig. 3A) showed a symmetric distribution on either side of the middle line, but an asymmetric distribution for the DAs group. Based on the funnel plot, some degree of publication bias was found in the DAs group (Supplementary Fig. 3B).

**Table 2:** Subgroup analysis of the biochemical cure rate in patients treated with DAs and surgery treatment

	DAs			Surgery		
	Pooled result	Number of studies	Number of patients	Pooled result	Number of studies	Number of patients
**Total**	0.78 (0.75, 0.82)	74	2659	0.66 (0.62, 0.71)	**81**	**4397**
**Microprolactinoma**	0.86 (0.78, 0.94)	9	238	0.79 (0.72, 0.85)	**23**	**686**
**Macroprolactinoma**	0.77 (0.72, 0.83)	27	1228	0.57 (0.46, 0.68)	**15**	**666**
**Giant prolactinoma**	0.62 (0.51, 0.74)	8	176	0.35 (0.08, 0.62)	**3**	**55**
**1980–1989**	0.74 (0.59, 0.89)	6	106	0.63 (0.52, 0.73)	**15**	**1134**
**1990–1999**	0.83 (0.75, 0.90)	11	397	0.64 (0.46, 0.83)	**7**	**262**
**2000–2009**	0.79 (0.72, 0.86)	18	605	0.69(0.60, 0.78)	**20**	**947**
**2010–2019**	0.77 (0.71, 0.82)	39	1551	0.67 (0.60, 0.74)	**39**	**2054**
**Bromocriptine**	0.70 (0.60, 0.80)	14	330	NA	NA	NA
**Cabergoline**	0.83 (0.78, 0.87)	32	1368	NA	NA	NA
**Microscopic surgery**	NA	NA	NA	0.68 (0.56, 0.80)	14	1043
**Endoscopic surgery**	NA	NA	NA	0.72 (0.65, 0.79)	29	1156

*Das* dopamine agonists, *NA* not applicable, because the data was not discussed or calculated in the meta-analysis

Cumulative meta-analysis was also conducted to detect the changes in the biochemical cure rate over time. Results showed an overall increasing trend of the biochemical cure rate of surgery, and after the year 2000, the biochemical cure rate of endoscopic surgery was consistently higher than that of bromocriptine (Fig. 4A).

### Recurrence rate

**Fig. 3:**
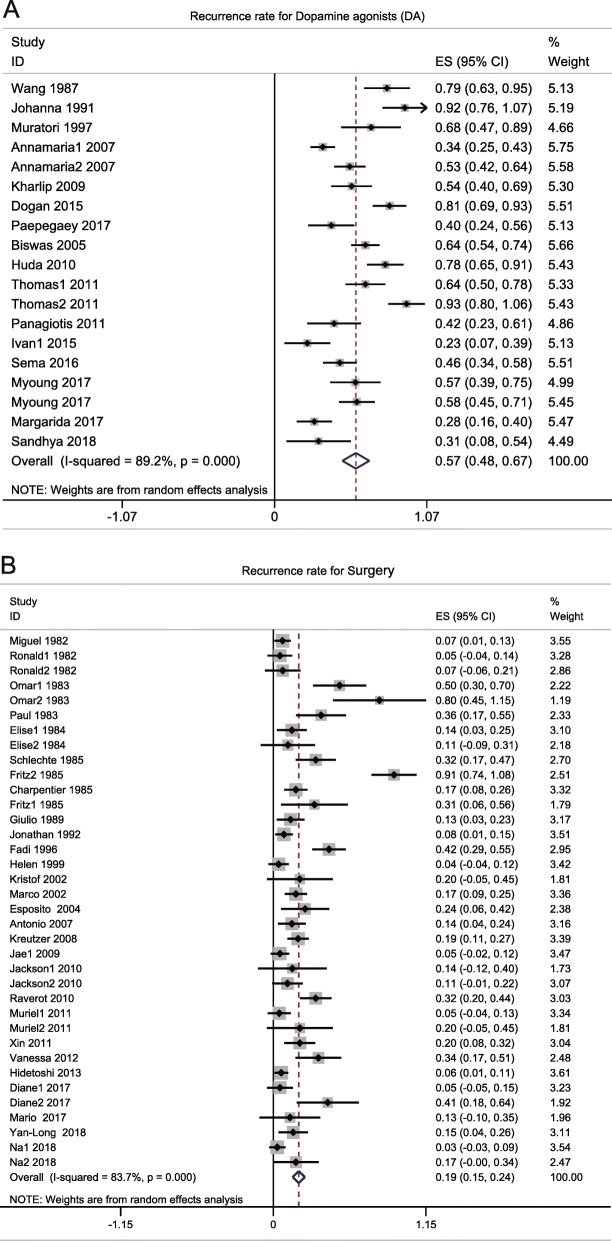
Forest plot for recurrence rate in prolactinoma patients treated with DAs (**a**) and patients treated with surgery (**b**)

This part consisted of 36 studies [4, 6, 93, 100, 102, 105, 111, 112, 114, 116, 120–122, 125, 127, 128, 132, 135, 138, 139, 141, 142, 145, 146, 148, 150, 154–156] comprising 1215 patients who underwent surgery and 19 studies [24, 27, 34, 39, 41, 47, 59, 62, 64, 68, 75, 82, 84, 85, 87] comprising 835 patients who used DAs. The recurrence rate of surgery was 0.19 (0.15, 0.24) (*I*^2^ = 83.7%, *p* = 0.000) and 0.57 (0.48, 0.67) (*I*^2^ = 89.2%, *p* = 0.000) for DAs (Fig. 3). Because of the high heterogeneity in surgery and DAs, subgroup analysis was carried out based on decades, subtypes of prolactinoma, subtypes of surgery, and drug species (Table 3; Supplementary Fig. 4). The following significant decreases in heterogeneity were detected: 2000–2009 (*I*^2^ = 47.1%, *p* = 0.093), microprolactinoma (*I*^2^ = 65.6%, *p* = 0.002), microscopic surgery (*I*^2^ = 65.7%, *p* = 0.020), and endoscopic surgery (*I*^2^ = 0.0%, *p* = 0.865) for surgery and bromocriptine (*I*^2^ = 15.5%, *p* = 0.277) for DAs (Table 3). Meta-regression analysis did not detect any important factors with respect to heterogeneity sources (Table 4).

**Table 3:** Subgroup analysis of the recurrence rate in patients treated with DAs and surgery treatment

	DAs			Surgery		
	Pooled result	Number of studies	Number of patients	Pooled result	Number of studies	Number of patients
**Total**	0.57 (0.48, 0.67)	19	835	0.19 (0.15, 0.24)	36	1215
**Microprolactinoma**	0.63 (0.49, 0.78)	7	380	0.10 (0.04, 0.17)	10	206
**Macroprolactinoma**	0.60 (0.39, 0.81)	6	226	0.34 (0.11, 0.56)	8	112
**Giant prolactinoma**	NA^a^	NA^a^	NA^a^	NA^a^	NA^a^	NA^a^
**1980–1989**	0.79	1	24	0.28 (0.16, 0.39)	13	374
**1990–1999**	0.81 (0.58, 1.04)	2	31	0.17 (− 0.01, 0.35)	3	149
**2000–2009**	0.51 (0.37, 0.65)	4	329	0.15 (0.09, 0.21)	6	278
**2010–2019**	0.54 (0.41, 0.67)	12	451	0.15 (0.09, 0.20)	14	414
**Bromocriptine**	0.86 (0.73, 0.98)	2	36	NA^b^	NA^b^	NA^b^
**Cabergoline**	0.55 (0.39, 0.70)	6	336	NA^b^	NA^b^	NA^b^
**Microscopic surgery**	NA^b^	NA^b^	NA^b^	0.13 (0.05, 0.21)	5	177
**Endoscopic surgery**	NA^b^	NA^b^	NA^b^	0.13 (0.05, 0.21)	3	75

*Das* dopamine agonists, *NA*^a^ not applicable, because the data was not provided by included studies, *NA*^b^ not applicable, because the data was not discussed or calculated in the meta-analysis

**Table 4:** Meta-regressiosn analysis of the biochemical cure rate and recurrence rate of DAs and surgery

	Biochemical cure rate		Recurrence rate	
	Surgery	DAs	Surgery	DAs
**Gender**	**0.019**	0.601	0.479	NA^a^
**Year**	0.154	0.103	0.479	NA^a^
**Age**	0.065	0.495	0.999	0.313
**Microprolactinoma**	0.880	0.578	0.350	0.732
**Macroprolactinoma**	**0.001**	0.235	0.068	0.836
**Giant prolactinoma**	0.482	**0.029**	NA^a^	NA^a^
**Microscopic surgery**	0.843	NA^b^	NA^a^	NA^b^
**Endoscopic surgery**	0.199	NA^b^	0.773	NA^b^
**Bromocriptine**	NA^b^	**0.024**	NA^b^	0.248
**Cabergoline**	NA^b^	0.935	NA^b^	0.520

*Das* dopamine agonists, *NA*^a^ not applicable, because the data was not provided by included studies or enough to be included in the meta-regression analysis. *NA*^b^ not applicable, because the data was not discussed or calculated in the meta-analysis

**Fig. 4:**
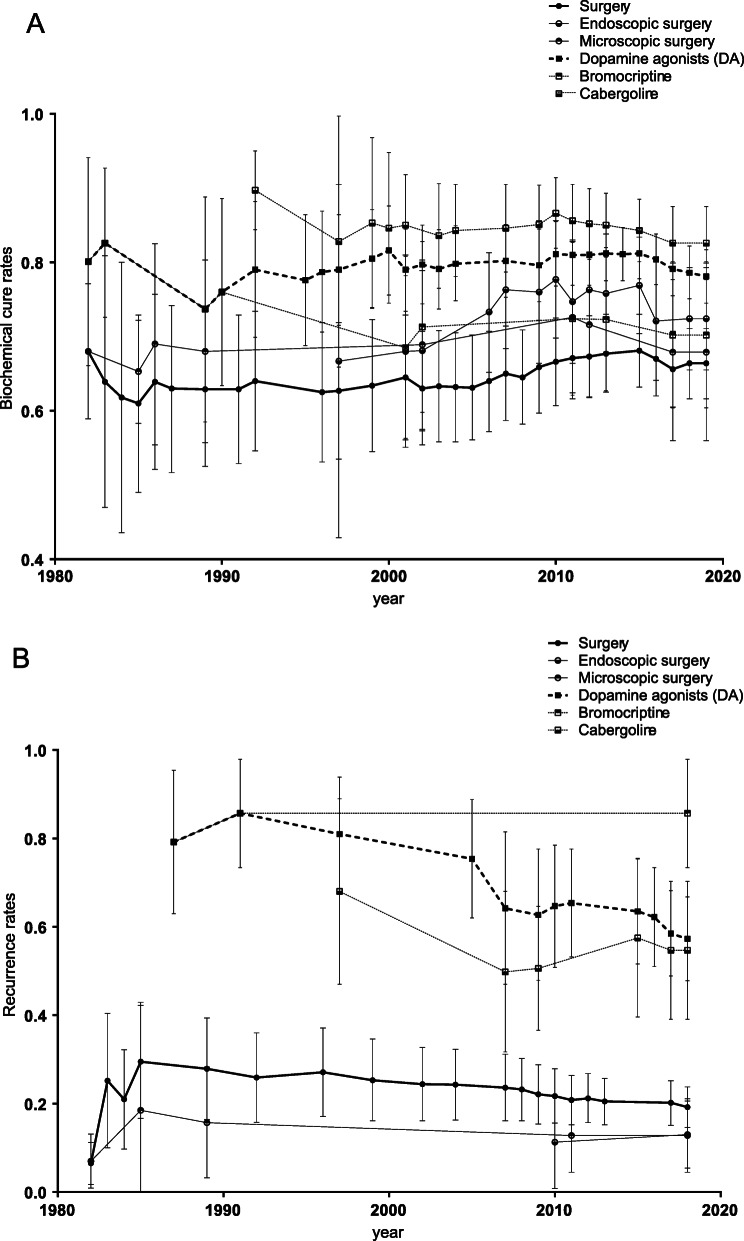
Cumulative meta-analysis of the biochemical cure rate (**a**) and recurrence rate (**b**) in prolactinoma patients subgrouped by the treatment methods

Cumulative meta-analysis of recurrence rates was carried out. Results showed that the recurrence rate of DAs decreased from 0.86 (0.73, 1.00) in 1991 to 0.57 (0.48, 0.67) in 2018. In the surgery group, the recurrence rate consistently reduced from 0.29 (0.15, 0.43) in 1985 to 0.18 (0.14, 0.21) in 2018 (Fig. 4B).

### Prolactin level

A total of 8 studies [7, 98, 124, 134, 150] comprising 555 patients in the surgery group and 27 studies [7, 31, 33, 38, 40, 42–44, 46, 48, 54, 55, 59, 78, 81, 83, 84, 90] comprising 954 patients in the DAs group were included in this part of research. Based on the pooled results, the mean differences in the prolactin levels between pre- and post-treatment were 396.80 ng/ml (222.33, 571.27) (*I*^2^ = 99%, *p* < 0.001) for surgery and 375.26 ng/ml (316.21, 434.31) (*I*^2^ = 98%, *p* < 0.001) for DAs (Supplementary Fig. 5). Sensitive analysis was conducted to find the source of heterogeneity, but no notable decrease in heterogeneity was detected.

### Symptom improvement rate

#### Improvement rate for vision impairment

In the surgery group, 114 patients from 11 studies [13, 95, 97, 124, 132, 137, 141, 143, 156] were included, and the pooled improvement rate for vision impairment was 0.68 (0.51, 0.82) (*I*^2^ = 34.8%, *p* = 0.018) (Table 5) with moderate heterogeneity. In the DAs group, 14 studies [5, 13, 29, 30, 33, 43, 46, 48, 71, 79] comprising 176 patients provided the required data, and the pooled improvement rate for vision impairment was 0.57 (0.38, 0.74) (*I*^2^ = 42.4%, *p* = 0.000) (Table 5; Supplementary Fig. 6A,7A) with moderate heterogeneity.

**Table 5:** The pooled estimated rate of symptom relief and the incidence rate of complications in DAs- and surgery-treated patients

	DAs			Surgery		
	Pooled result	Number of studies	Number of patients	Pooled result	Number of studies	Number of patients
**Vision impairment improvement rate**	0.57 (0.38, 0.74)	14	176	0.68 (0.51, 0.82)	**11**	**114**
**Headache improvement rate**	0.86 (0.72, 0.94)	4	35	0.80 (0.32, 0.97)	3	95
**Menstrual disturbance improvement rate**	0.71 (0.16, 0.97)	6	123	0.68 (0.62, 0.74)	3	226
**Galactorrhoea improvement rate**	0.89 (0.72, 0.96)	6	29	0.33 (0.01, 0.94)	**3**	**176**
**Incidence rate of ACTH insufficiency**	0.10 (0.06, 0.16)	9	286	0.25 (0.13, 0.43)	**11**	**387**
**Incidence rate of TSH deficiency**	0.19 (0.12, 0.28)	7	194	0.24 (0.14, 0.38)	12	475
**Incidence rate of hypopituitarism**	0.29 (0.13, 0.54)	4	99	0.17 (0.06, 0.38)	**11**	**709**
**Incidence rate of diabetes insipidus**	NA	NA	NA	0.17 (0.12, 0.25)	27	1616

*Das* dopamine agonists, *NA* not applicable, because the data was not provided by included studies

#### Headache improvement rate

A total of 3 studies [95, 98, 132] comprising 95 patients treated with surgery were included, and the pooled headache improvement rate was 0.80 (0.32, 0.97) (*I*^2^ = 46.9%, *p* = 0.000). Meta-analysis of this part was conducted for DAs using 35 patients from 4 studies [5, 30, 32, 46]. The pooled headache improvement rate of DAs was 0.86 (0.72, 0.94) (*I*^2^ = 0%, *p* = 0.416) with low heterogeneity (Table 5; Supplementary Fig. 6B,7B).

#### Improvement rate for menstrual disturbance

A total of 3 studies [94, 141, 154] comprising 226 patients treated with surgery and 6 studies [20, 28, 30, 71] comprising 123 patients who used DAs were included, and the pooled improvement rates for menstrual disturbance were 0.68 (0.62, 0.74) (*I*^2^ = 0%, *p* = 0.327) and 0.71 (0.16, 1.00) (*I*^2^ = 47.5%, *p* = 0.000), respectively (Table 5; Supplementary Fig. 6C,7C).

#### Galactorrhoea improvement rate

This research included 3 studies [124, 132, 141] comprising 176 patients treated with surgery and 6 studies [30, 32, 43, 71] comprising 29 patients who used DAs to assess the galactorrhoea improvement rate after these treatments. The pooled galactorrhoea improvement rates were 0.33 (0.01, 0.94) (*I*^2^ = 47.1%, *p* = 0.000) after surgery and 0.89 (0.72, 0.96) (*I*^2^ = 0%, *p* = 0.493) after DAs, respectively (Table 5; Supplementary Fig. 6D,7D).

### Complications

#### Incidence rate of ACTH insufficiency

A total of 387 patients from 11 studies [3, 5, 6, 13, 93, 98, 121, 151, 152, 154] that applied surgery and 286 patients from 9 studies [3, 5, 13, 33, 45, 73, 78] that utilized DAs were included, and the pooled incidence rates of ACTH insufficiency were 0.25 (0.13, 0.43) (*I*^2^ = 46.7%, *p* = 0.000) for surgery and 0.10 (0.06, 0.16) (*I*^2^ = 26.0%, *p* = 0.121) for DAs, respectively (Table 5; Supplementary Fig. 6E,7E).

#### Incidence rate of TSH deficiency

In this part, 12 studies [3–6, 13, 93, 98, 151, 152, 154] comprising 475 patients who underwent surgery and 7 studies [3, 5, 13, 23, 61, 73, 88] comprising 194 DAs-treated patients were included, and the pooled estimated rates were 0.24 (0.14, 0.38) (*I*^2^ = 45.4%, *p* = 0.000) and 0.19 (0.12, 0.28) (*I*^2^ = 26.4%, *p* = 0.134) after surgery and DAs, respectively (Table 5; Supplementary Fig. 6F,7F).

#### Incidence rate of hypopituitarism

A total of 709 surgery-treated patients from 11 studies [5, 6, 97, 124, 141, 147, 148, 156] and 99 DAs-treated patients from 4 studies [5, 48] were included to assess the incidence rate of hypopituitarism. The pooled incidence rates were 0.17 (0.06, 0.38) (*I*^2^ = 48.4%, *p* = 0.000) for surgery and 0.29 (0.13, 0.54) (*I*^2^ = 41.6%, *p* = 0.015) for DAs, respectively (Table 5; Supplementary Fig. 6G,7G).

#### Incidence rate of diabetes insipidus

Because of the lack of studies that used DAs and reported the incidence rate of diabetes insipidus, only 1616 surgery-treated patients from 27 studies [3–5, 93, 98, 99, 115, 117, 124, 126, 132, 138, 140, 141, 143, 145, 147–154, 156] were included to detect the pooled incidence rate. The estimated incidence rate of diabetes insipidus after surgery was 0.17 (0.12, 0.25) (*I*^2^ = 47.1%, *p* = 0.000) (Table 5; Supplementary Fig. 6H).

## Discussion

DAs are the preferred choice in the current guideline, and they are used for treating symptomatic microprolactinomas and macroprolactinomas [157]. Compared with DAs, surgery has very limited indications, which include the following: (1) intolerance or resistance to DAs; (2) acute complications such as pituitary apoplexy and cerebrospinal fluid leak [157]. Some new indications have been discussed in other papers, which include the following: (3) Young patients with high complete resection rate; (4) unwillingness to take long-term medication; (5) cystic prolactinoma; (6) partial resistance to treatment; and (7) requirement of high dose of cabergoline [158]. The reasons for these limited indications are a reported high recurrence rate (7–50%), possible complications, and requirement of experienced neurosurgeons [157].

Over the past 5 decades, the endoscope has developed from a diagnostic tool to a mature surgical technique with concepts of minimally invasive surgery and key-hole surgery [159]. An increasing number of neurosurgeons have accepted this vivifying technique and have promoted its indications. Based on our results, surgery, especially endoscopic surgery, has already shown satisfactory efficacy and safety in some subgroups of prolactinoma patients, and it is time to re-evaluate the surgical indications of prolactinoma.

### DAs versus surgery for microprolactinoma

Symptomatic microprolactinoma patients are recommended to receive DAs in the current guideline [157], although a microprolactinoma rarely grows. But the pooled estimated biochemical cure rate of endoscopic surgery was the same as that of DAs (0.86 versus 0.86) and it was slightly higher than that of bromocriptine (0.86 versus 0.76). Furthermore, the recurrence rates of surgery, both microscopic and endoscopic surgery, were much lower than those of DAs (0.10 versus 0.63). In another meta-analysis conducted by Ma et al. [10], the reported long-term remission rates for microprolactinoma were 56% (medication) versus 91% (surgery). The difference between their results and our results may have arisen from different inclusion criteria, as they excluded patients utilizing DAs before surgery. Zamanipoor et al. also conducted a meta-analysis and found the long-term remission rates were 36% versus 83% for medication and surgery separately(9). This may be due to that they only include patients with medicine withdrawal. It is notable that some countries like China do not allow the use of cabergoline, and patients living in such countries may consider surgery to be a better choice than bromocriptine.

### DAs versus surgery for macroprolactinoma

All macroprolactinoma patients with or without symptoms are recommended to use DAs [157]. The same preference was detected in our results, which showed that DAs had a higher biochemical cure rate than surgery (0.77 versus 0.57). However, some interesting results were also found in the subgroup analysis. The only one included microscopic study in the microsurgery group reported the highest biochemical cure rate. Furthermore, endoscopic surgery and bromocriptine were at the same level in terms of the biochemical cure rate (0.66 versus 0.64) and endoscopic surgery was lower than bromocriptine in terms of the recurrence rate (0.11 versus 0.92). Results for the long-term remission rates in the study by Ma et al. [10] showed a similar tendency to that in our study (77% versus 44%). But the results from Zamanipoor et al. showed that the long-term remission rates were 28% versus 60% for medication and surgery separately [9]. The difference between their results and ours may come from that they only include patients with medication withdrawal.

### DAs versus surgery for giant prolactinoma

For giant prolactinoma, we failed to include studies reporting the biochemical cure rate after microscopic surgery or bromocriptine and the recurrence rate after any treatment. This may be because of our strict inclusion criteria, as we excluded studies with less than 10 patients or studies using another treatment like radiotherapy. In our results, DAs showed a higher biochemical cure rate than surgery (0.62 versus 0.35). Similar but exaggerated results were reported by Lv et al. [13] (0.48 versus 0, DAs versus surgery). Hamidi et al. also detected similar remission rates (58.8% versus 53.6%, DAs versus surgery). Because of the lack of data from giant prolactinoma patients, no recommendations are found in the current guidelines. Further researches should address this question and verify our results in future guidelines.

### Comparison of relief of symptoms between DAs and surgery

A large prolactinoma can compress the surrounding structures and can cause severe vision impairment and headache [160], which are also the indications for surgery. Lv et al. [13] reported that DAs and surgery had a similar recovery rate for visual impairment. However, it is interesting that the current research reported a slightly higher improvement rate for vision impairment in surgery-treated patients (0.68 versus 0.57) and a comparable headache improvement rate in DAs-treated patients (0.80 versus 0.86); thus, showing that surgery and DAs may have a similar ability in relieving nerve compression.

We found preference of DAs in terms of the improvement rate for menstrual disturbance (0.71 versus 0.68) and galactorrhea (0.89 versus 0.33). Nayan et al. [11] conducted a meta-analysis on the fertility after surgery in prolactinoma patients, and they reported a significant decrease in the pooled prevalence of galactorrhea from 84 to 29%. The reduction was greater than that in our study, which may have been caused by gender restriction in the inclusion criteria.

### Comparison of the rate of complications between DAs and surgery

A low rate of complications was noted for both treatments. Our results revealed a preference for DAs in ACTH insufficiency (0.10 versus 0.25) and TSH deficiency (0.19 versus 0.24) but a higher incidence rate of hypopituitarism (0.29 versus 0.17) after DAs. Oksana et al. [5] reported similar results in ACTH insufficiency and TSH deficiency but a contrary result in hypopituitarism, and all of the results from their study were higher than our results (ranging from 27 to 69%). A different population, as they only included giant prolactinoma cases, may explain this discrepancy.

The incidences of diabetes insipidus in different studies range from 2.5 to 100%, with the pooled result being 0.174 (0.118, 0.251). Because no studies on DAs-treated patients with diabetes insipidus were included, we failed to compare the outcome between DAs and surgery.

### Comparison of the cost of therapy between DAs and surgery

The cost of DAs and surgery is a complex consideration, and contrary results have been reported. Lian et al. [161] reported that for microprolactinoma patients, the estimated costs of surgery and DAs were ¥22,527 and ¥20,555. For macroprolactinoma patients, the estimated costs were ¥42,357/¥44,094 in males/females for surgery and ¥31,461/¥27,178 in males/females for DAs. Similar results were found by Zhen et al. [162]. But Corinna et al. [163] reported different results; they reported that the lifetime costs of surgery, bromocriptine, and cabergoline were $40,473, $41,601, and $70,696, respectively. Further studies are needed to determine which method is more cost-effective.

### DAs treatment before surgery?

In the current research, we conducted subgroup analysis for surgery treated population based on DAs treatment history and found similar normalization rates between patients with DAs treatment history (0.66) and without DAs treatment history (0.69; Supplementary Fig. 8). This result showed that DAs treatment before surgery may not influence the efficiency of surgery. Because all included researches for the safety analysis only discussed patients with DAs treatment history or provided inseparable data of these two situations, we did not explore the difference of surgery safety between patients with or without DAs treatment history.

### Duration of medication

The mean duration of medication treatment in the DAs treatment group was 44.5 months. But most studies defined resistance to DA as a lack of PRL normalization and a failure to decrease tumor size despite an adequate dose of DA treatment for 3 or 6 months [99, 127]. For patients who were resistant to DAs treatment, they were recommended to increase the dose to maximal tolerable doses [157]. And for patients who have no response to DAs, they were recommended to accept transsphenoidal surgery [157].

### Advantages and limitations

As this was the first study to compare the efficacy and safety between DAs and surgery in patients with all types of prolactinomas, we included a large sample size of up to 6162 patients.

The major limitation of the present research was that we could not perform a two-arm meta-analysis due to the lack of prospective randomized controlled trials. We could only collect the data from single-arm studies. And because of the different indications for surgery and DAs, the patient groups differed significantly between each other. So, we conducted qualitative comparison between treatments instead of a quantitative comparison in the current meta-analysis. Randomized controlled trials of DAs and surgery are expected in the future.

Another limitation was the high heterogeneity of the biochemical cure rate and the recurrence rate. Although we conducted a subgroup analysis and a meta-regression analysis to identify the source of heterogeneity, we only found that giant prolactinoma and bromocriptine could partially explain the heterogeneity. We failed to collect the following data and proceed with a comparison of the following parts: biochemical cure rate in giant prolactinoma patients using microscopic surgery or bromocriptine, recurrence rate in all giant prolactinoma patients, recurrence rate in microprolactinoma patients treated with bromocriptine, and incidence rate of diabetes insipidus in DAs-treated patients. The lack of data may have arisen from our inclusion criteria of patient size limitation. Most DAs withdrawal studies focused on cabergoline, and few studies on bromocriptine were excluded from this research because of our exclusion criteria. Further clinical researches on these patients are needed.

The present study did not include the radiological parameters of prolactinoma. Further researches are needed to verify our results.

## Conclusion

The present meta-analysis serves as the first study to compare the efficacy and safety between DAs and surgery in microprolactinoma and macroprolactinoma patients. We concluded that for patients with clear indications or contraindications for surgery, choosing surgery or DAs accordingly is unequivocal. However, for patients with clinical equipoise, further controlled clinical trials are expected to address it. In this meta-analysis, we discovered that surgery, especially endoscopic surgery, showed comparable efficacy and safety in microprolactinoma and macroprolactinoma patients with a considerable biochemical cure rate, lower recurrence rate, and similar improvement rates of symptoms and incidence rates of complications. With the development of surgical technique and equipment, the efficacy and safety of surgery have greatly improved. Therefore, we suggest that neurosurgeons and endocrinologists conduct high-quality clinical trials to address the clinical equipoise quantitatively.

## Supplementary Information

**Additional file 1: Supplementary Figure 1**. A. Summary of Risk of bias assessment for randomized controlled trials using ROB.2 tool. B. Summary of Risk of Bias assessment for non-randomized controlled trials using ROBINS-I tool.

**Additional file 2: Supplementary Figure 2.** Forest plots for subgroup analysis of biochemical cure rates in surgery-treated patients subgrouped by patients type (A), publication years (B), surgery types (C); and in DAs-treated patients subgrouped by patients type (D), publication years (E), DAs types (F).

**Additional file 3: Supplementary Figure 3.** Funnel plots for biochemical cure rate of patients treated with surgery (A) and DAs (B).

**Additional file 4: Supplementary Figure 4.** Forest plots for subgroup analysis of recurrence rates in surgery-treated patients subgrouped by patients type (A), publication years (B), surgery types (C); and in DAs-treated patients subgrouped by patients type (D), publication years (E), DAs types (F).

**Additional file 5: Supplementary Figure 5.** Forest plots for prolactin level of patients applying surgery (A) and DAs (B).

**Additional file 6: Supplementary Figure 6.** Forest plots for improvement rates for vision impairment (A), headache (B), menstrual disturbance (C), galactorrhoea (D) and incidence rates of ACTH insufficiency (E), TSH deficiency (F), hypopituitarism (G), diabetes insipidus (H) of patients applying surgery.

**Additional file 7: Supplementary Figure 7.** Forest plots for improvement rates for vision impairment (A), headache (B), menstrual disturbance (C), galactorrhoea (D) and incidence rates of ACTH insufficiency (E), TSH deficiency (F), hypopituitarism (G) of patients applying DAs.

**Additional file 8: Supplementary Figure 8.** Forest plots for subgroup analysis of biochemical cure rates in surgery-treated patients subgrouped by DAs treatment history.

**Additional file 9: Supplementary Table 1.** Basic characteristics of the included studies with surgery treatment.

**Additional file 10: Supplementary Table 2.** Basic characteristics of the included studies with DAs treatment.

**Additional file 11: Supplementary Table 3.** Summary table of risk of bias for RCT.

**Additional file 12: Supplementary Table 4.** Summary table of risk of bias for non-RCT.

**Additional file 13: Supplementary Table 5.** Summary table of risk of bias for case-series study.

**Additional file 14: Supplementary file 1.** Literature research strategy.

## Abbreviations

ACTH: Adrenocorticotropic hormone
TSH: Thyroid-stimulating hormone
DAs: Dopamine agonists
RE: Random-effects
CAB: Cabergoline
BRC: Bromocriptine
NA: Not applicable
