# Supplementary figures and images for: Are dopamine agonists still the first-choice treatment for prolactinoma in the era of endoscopy? A systematic review and meta-analysis
Source: Chin Neurosurg J. 2022 Apr 8;8:9. doi: 10.1186/s41016-022-00277-1 (PMC8994364; doi:10.1186/s41016-022-00277-1)

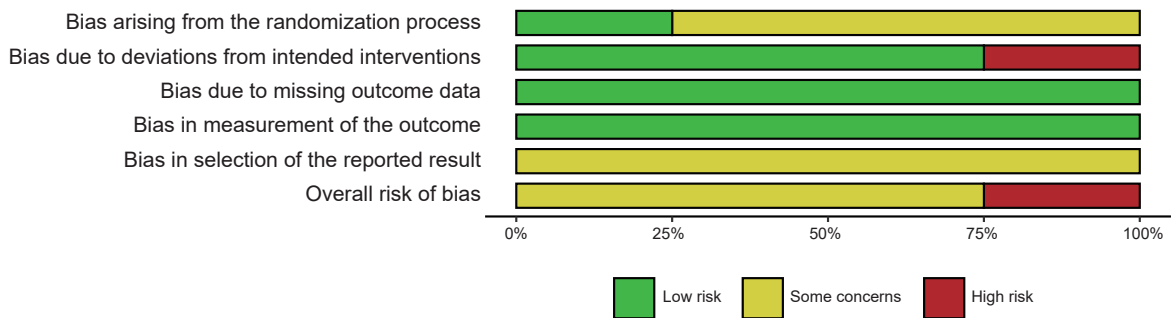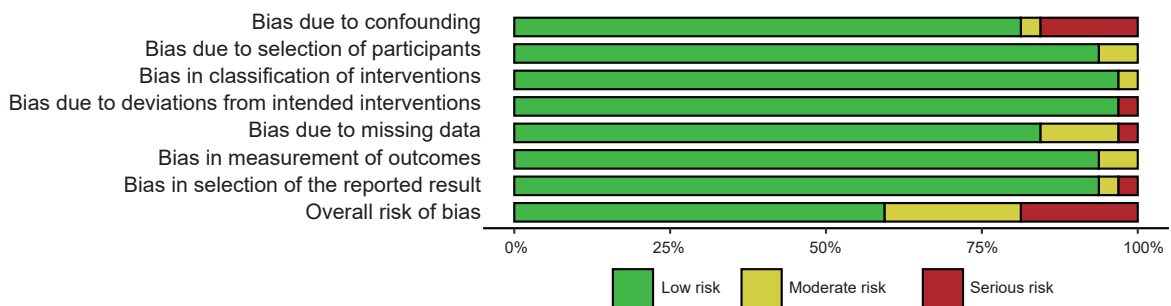

Supplement: Supplementary file 1 — Additional file 1: Supplementary Figure 1. A. Summary of Risk of bias assessment for randomized controlled trials using ROB.2 tool. B. Summary of Risk of Bias assessment for non-randomized controlled trials using ROBINS-I tool. [file 41016_2022_277_MOESM1_ESM.pdf]

**A**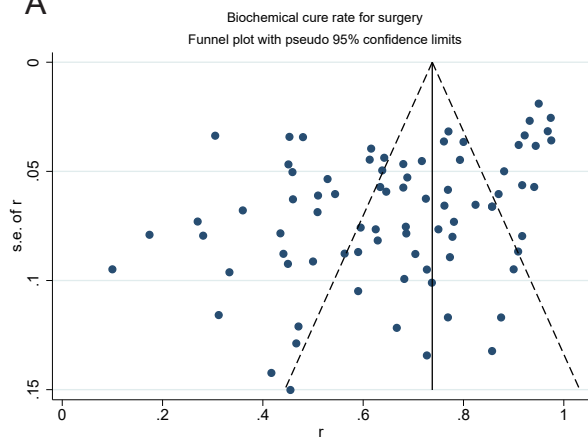**B**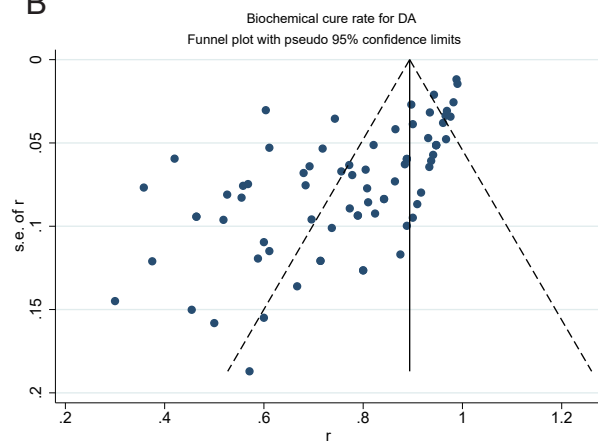

Supplement: Supplementary file 3 — Additional file 3: Supplementary Figure 3. Funnel plots for biochemical cure rate of patients treated with surgery (A) and DAs (B). [file 41016_2022_277_MOESM3_ESM.pdf]

A

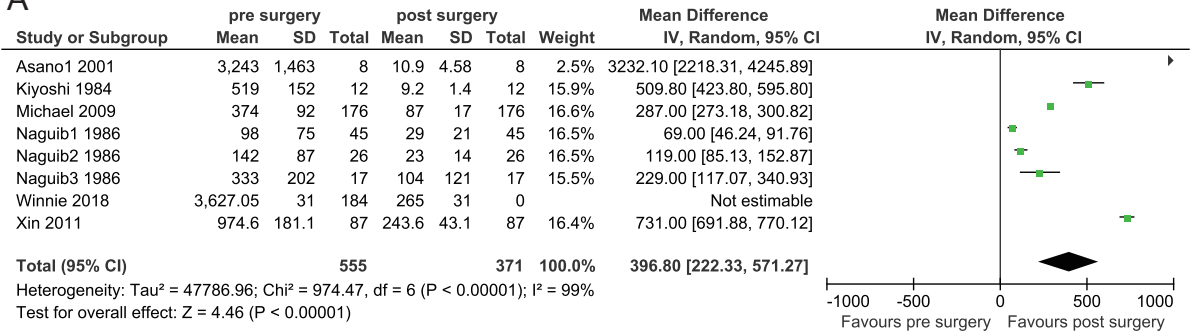

B

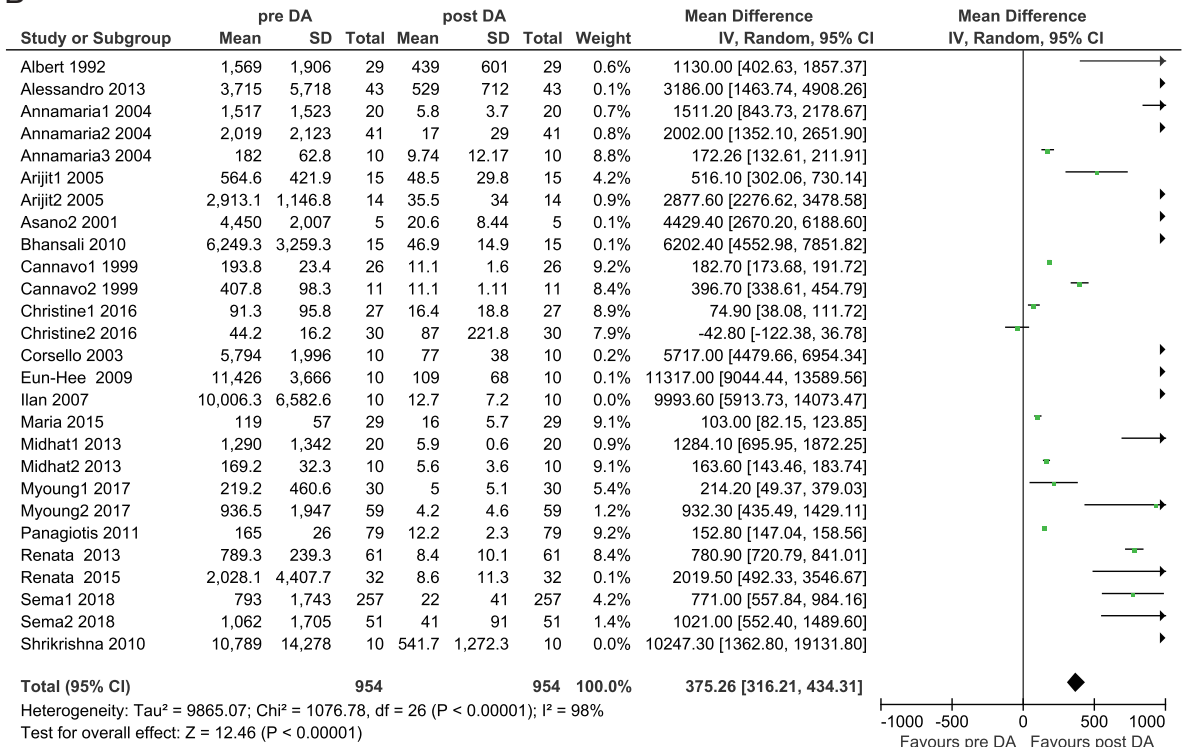

Supplement: Supplementary file 5 — Additional file 5: Supplementary Figure 5. Forest plots for prolactin level of patients applying surgery (A) and DAs (B). [file 41016_2022_277_MOESM5_ESM.pdf]

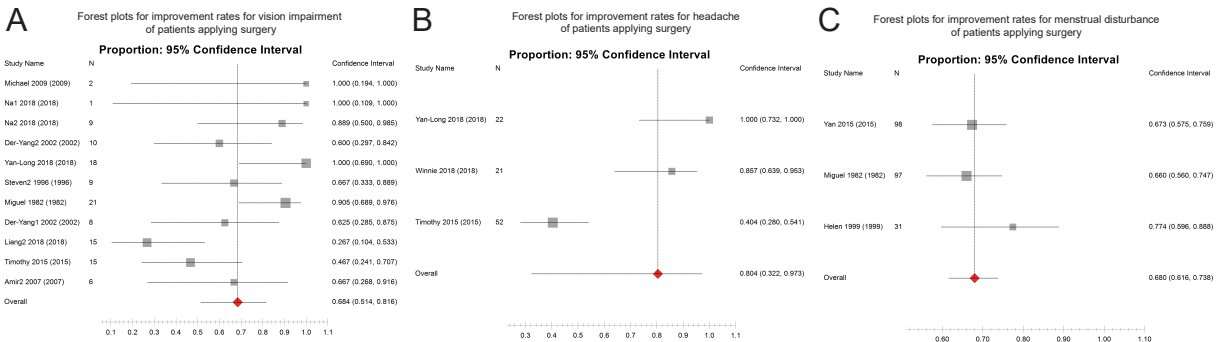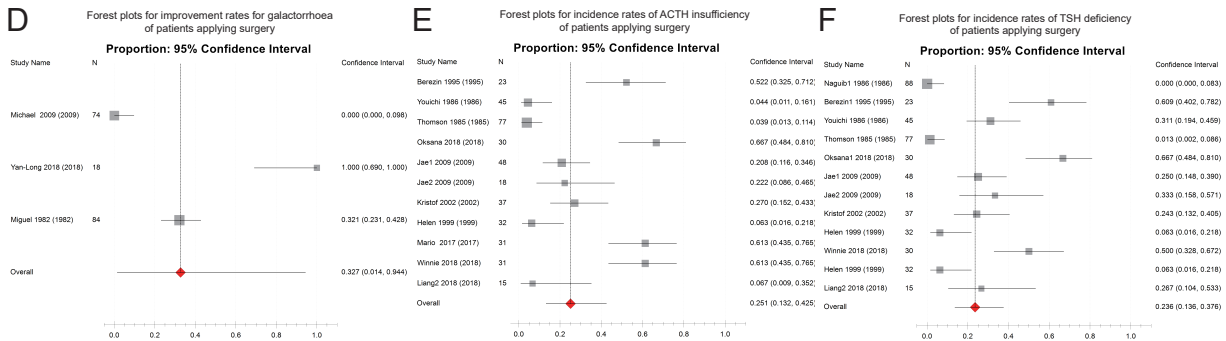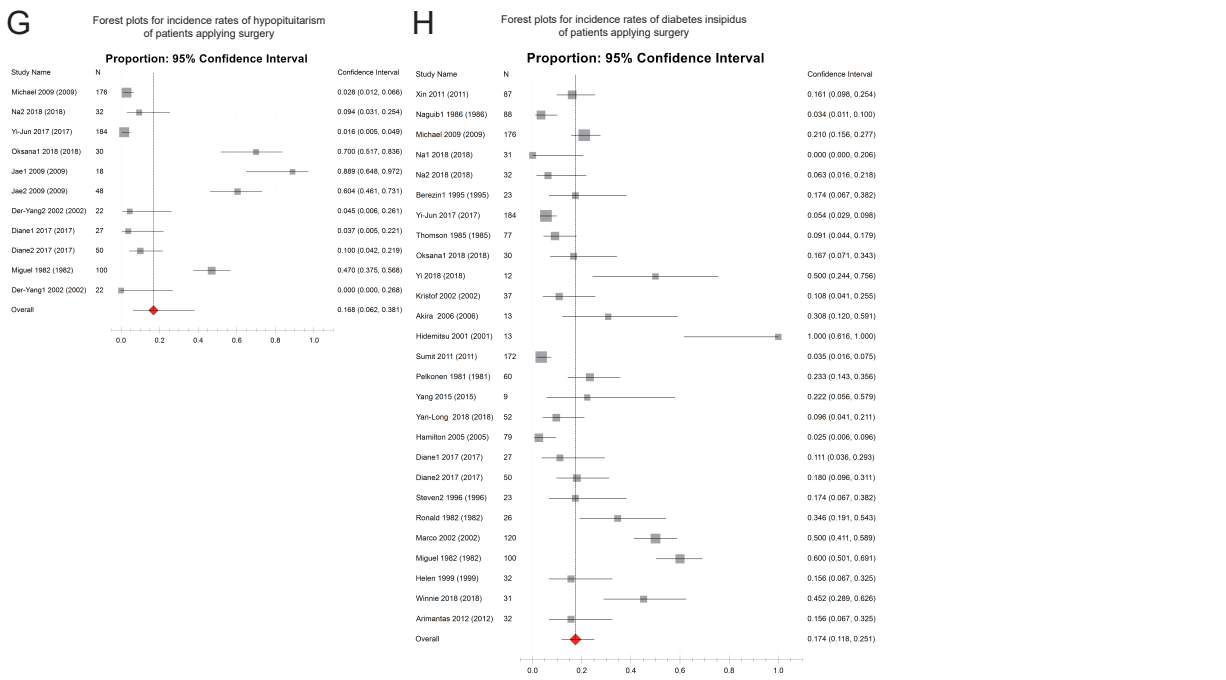

Supplement: Supplementary file 6 — Additional file 6: Supplementary Figure 6. Forest plots for improvement rates for vision impairment (A), headache (B), menstrual disturbance (C), galactorrhoea (D) and incidence rates of ACTH insufficiency (E), TSH deficiency (F), hypopituitarism (G), diabetes insipidus (H) of patients applying surgery. [file 41016_2022_277_MOESM6_ESM.pdf]

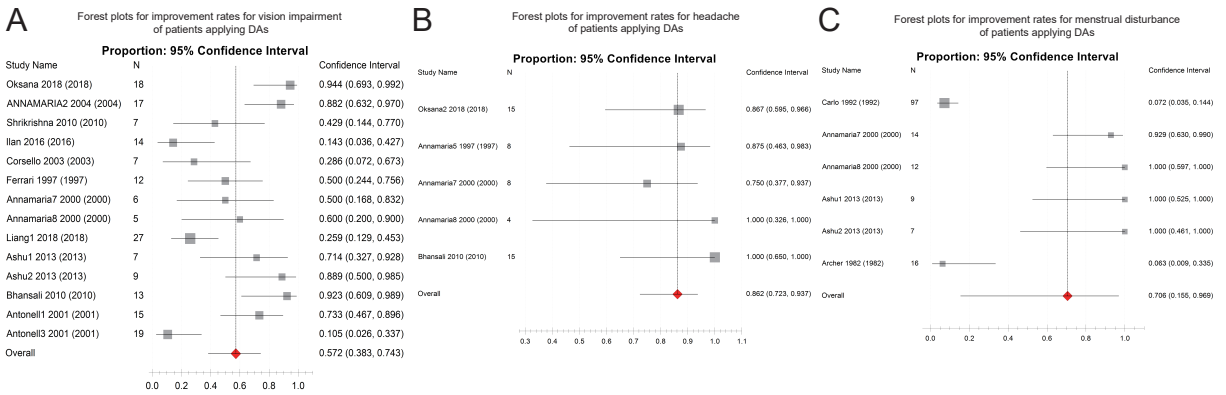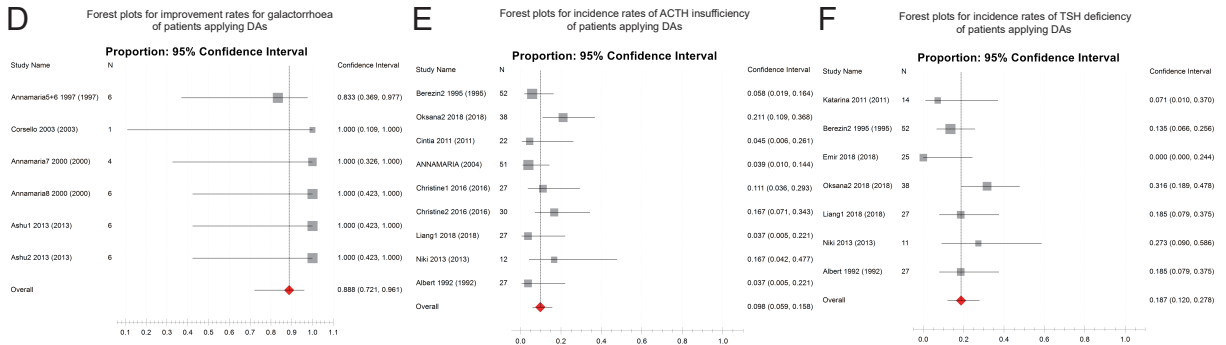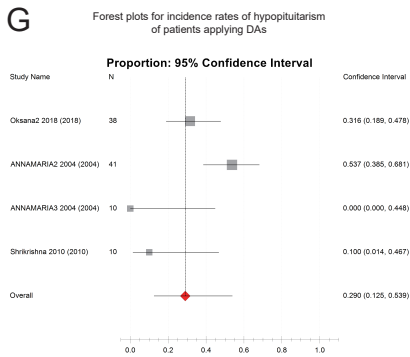

Supplement: Supplementary file 7 — Additional file 7: Supplementary Figure 7. Forest plots for improvement rates for vision impairment (A), headache (B), menstrual disturbance (C), galactorrhoea (D) and incidence rates of ACTH insufficiency (E), TSH deficiency (F), hypopituitarism (G) of patients applying DAs. [file 41016_2022_277_MOESM7_ESM.pdf]

Biochemical cure rate for surgery (subgrouped by DAs treatment history)

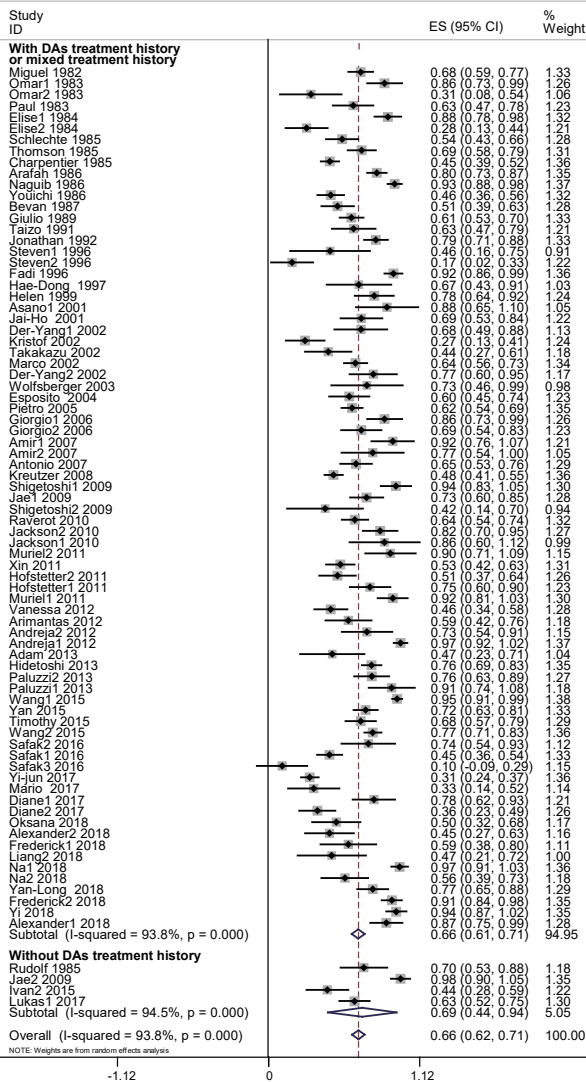

Supplement: Supplementary file 8 — Additional file 8: Supplementary Figure 8. Forest plots for subgroup analysis of biochemical cure rates in surgery-treated patients subgrouped by DAs treatment history. [file 41016_2022_277_MOESM8_ESM.pdf]
