# Supplementary material for: Are dopamine agonists still the first-choice treatment for prolactinoma in the era of endoscopy? A systematic review and meta-analysis
Source: Chin Neurosurg J. 2022 Apr 8;8:9. doi: 10.1186/s41016-022-00277-1 (PMC8994364; doi:10.1186/s41016-022-00277-1)
Supplement: Supplementary file 4 — Additional file 4: Supplementary Figure 4. Forest plots for subgroup analysis of recurrence rates in surgery-treated patients subgrouped by patients type (A), publication years (B), surgery types (C); and in DAs-treated patients subgrouped by patients type (D), publication years (E), DAs types (F). [file 41016_2022_277_MOESM4_ESM.pdf]

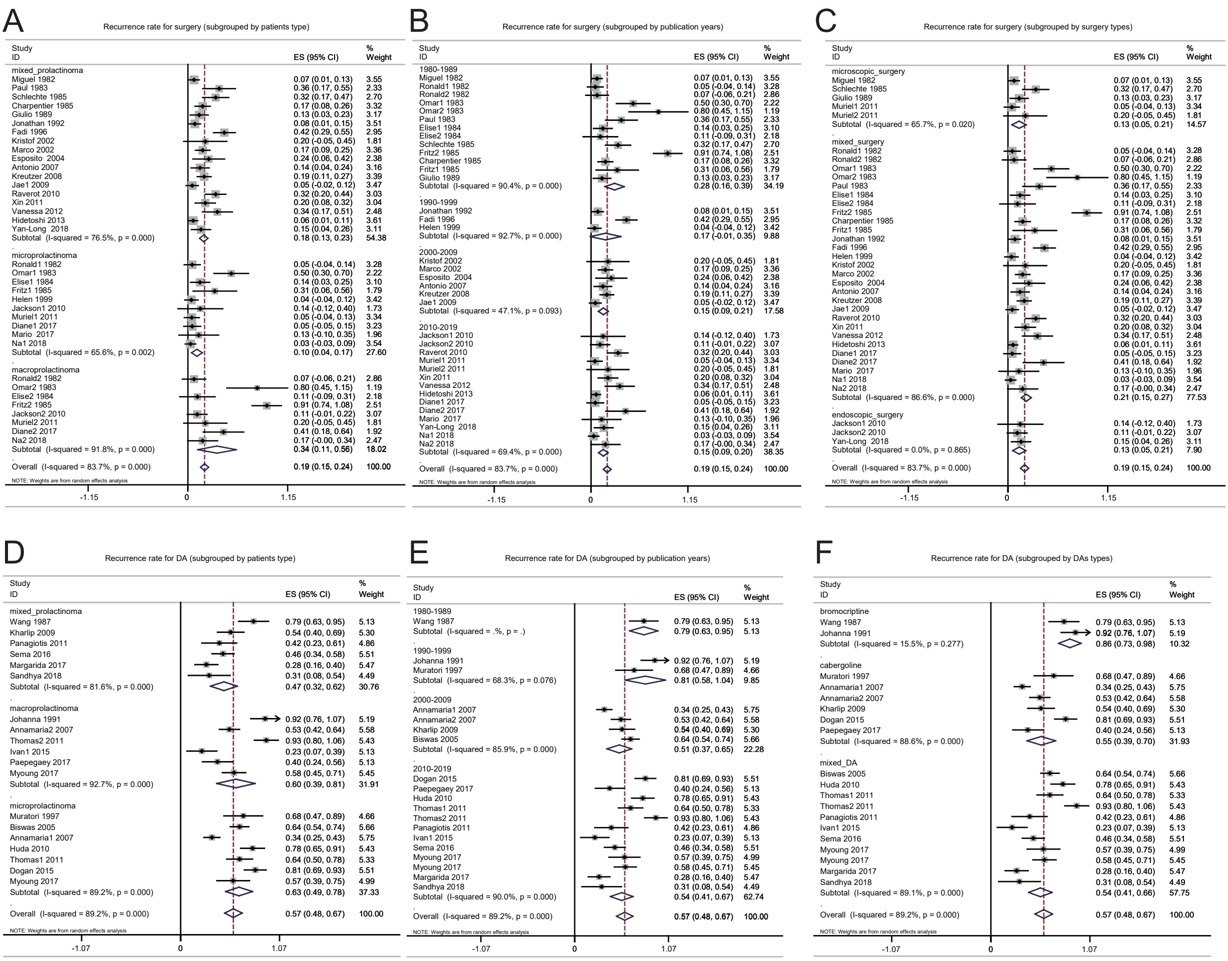
A
Recurrence rate for surgery (subgrouped by patients type)
Study ID | ES (95% CI) | % Weight
mixed_prolactinoma
Miguel 1982 0.07 (0.01, 0.13) 3.55
Paul 1983 0.36 (0.17, 0.55) 2.33
Schlechte 1985 0.32 (0.17, 0.47) 2.70
Charpentier 1985 0.17 (0.08, 0.26) 3.32
Giulio 1989 0.13 (0.03, 0.23) 3.17
Jonathan 1992 0.08 (0.01, 0.15) 3.51
Fadi 1996 0.42 (0.29, 0.55) 2.95
Kristof 2002 0.20 (-0.05, 0.45) 1.81
Marco 2002 0.17 (0.09, 0.25) 3.36
Esposito 2004 0.24 (0.06, 0.42) 2.38
Antonio 2007 0.14 (0.04, 0.24) 3.16
Kreutzer 2008 0.19 (0.11, 0.27) 3.39
Jae1 2009 0.05 (-0.02, 0.12) 3.47
Raverot 2010 0.32 (0.20, 0.44) 3.03
Xin 2011 0.20 (0.08, 0.32) 3.04
Vanessa 2012 0.34 (0.17, 0.51) 2.48
Hidetoshi 2013 0.06 (0.01, 0.11) 3.61
Yan-Long 2018 0.15 (0.04, 0.26) 3.11
Subtotal (I-squared = 76.5%, p = 0.000) 0.18 (0.13, 0.23) 54.38
microprolactinoma
Ronald1 1982 0.05 (-0.04, 0.14) 3.28
Omar1 1983 0.50 (0.30, 0.70) 2.22
Elise1 1984 0.14 (0.03, 0.25) 3.10
Fritz1 1985 0.31 (0.06, 0.56) 1.79
Helen 1999 0.04 (-0.04, 0.12) 3.42
Jackson1 2010 0.14 (-0.12, 0.40) 1.73
Muriel1 2011 0.05 (-0.04, 0.13) 3.34
Diane1 2017 0.05 (-0.05, 0.15) 3.23
Mario 2017 0.13 (-0.10, 0.35) 1.96
Na1 2018 0.03 (-0.03, 0.09) 3.54
Subtotal (I-squared = 65.6%, p = 0.002) 0.10 (0.04, 0.17) 27.60
macroprolactinoma
Ronald2 1982 0.07 (-0.06, 0.21) 2.86
Omar2 1983 0.80 (0.45, 1.15) 1.19
Elise2 1984 0.11 (-0.09, 0.31) 2.18
Fritz2 1985 0.91 (0.74, 1.08) 2.51
Jackson2 2010 0.11 (-0.01, 0.22) 3.07
Muriel2 2011 0.20 (-0.05, 0.45) 1.81
Diane2 2017 0.41 (0.18, 0.64) 1.92
Na2 2018 0.17 (-0.00, 0.34) 2.47
Subtotal (I-squared = 91.8%, p = 0.000) 0.34 (0.11, 0.56) 18.02
Overall (I-squared = 83.7%, p = 0.000) 0.19 (0.15, 0.24) 100.00
NOTE: Weights are from random effects analysis
-1.15 0 1.15
B
Recurrence rate for surgery (subgrouped by publication years)
Study ID | ES (95% CI) | % Weight
1980-1989
Miguel 1982 0.07 (0.01, 0.13) 3.55
Ronald1 1982 0.05 (-0.04, 0.14) 3.28
Ronald2 1982 0.07 (-0.06, 0.21) 2.86
Omar1 1983 0.50 (0.30, 0.70) 2.22
Omar2 1983 0.80 (0.45, 1.15) 1.19
Paul 1983 0.36 (0.17, 0.55) 2.33
Elise1 1984 0.14 (0.03, 0.25) 3.10
Elise2 1984 0.11 (-0.09, 0.31) 2.18
Schlechte 1985 0.32 (0.17, 0.47) 2.70
Fritz2 1985 0.91 (0.74, 1.08) 2.51
Charpentier 1985 0.17 (0.08, 0.26) 3.32
Fritz1 1985 0.31 (0.06, 0.56) 1.79
Giulio 1989 0.13 (0.03, 0.23) 3.17
Subtotal (I-squared = 90.4%, p = 0.000) 0.28 (0.16, 0.39) 34.19
1990-1999
Jonathan 1992 0.08 (0.01, 0.15) 3.51
Fadi 1996 0.42 (0.29, 0.55) 2.95
Helen 1999 0.04 (-0.04, 0.12) 3.42
Subtotal (I-squared = 92.7%, p = 0.000) 0.17 (-0.01, 0.35) 9.88
2000-2009
Kristof 2002 0.20 (-0.05, 0.45) 1.81
Marco 2002 0.17 (0.09, 0.25) 3.36
Esposito 2004 0.24 (0.06, 0.42) 2.38
Antonio 2007 0.14 (0.04, 0.24) 3.16
Kreutzer 2008 0.19 (0.11, 0.27) 3.39
Jae1 2009 0.05 (-0.02, 0.12) 3.47
Subtotal (I-squared = 47.1%, p = 0.093) 0.15 (0.09, 0.21) 17.58
2010-2019
Jackson1 2010 0.14 (-0.12, 0.40) 1.73
Jackson2 2010 0.11 (-0.01, 0.22) 3.07
Raverot 2010 0.32 (0.20, 0.44) 3.03
Muriel1 2011 0.05 (-0.04, 0.13) 3.34
Muriel2 2011 0.20 (-0.05, 0.45) 1.81
Xin 2011 0.20 (0.08, 0.32) 3.04
Vanessa 2012 0.34 (0.17, 0.51) 2.48
Hidetoshi 2013 0.06 (0.01, 0.11) 3.61
Diane1 2017 0.05 (-0.05, 0.15) 3.23
Diane2 2017 0.41 (0.18, 0.64) 1.92
Mario 2017 0.13 (-0.10, 0.35) 1.96
Yan-Long 2018 0.15 (0.04, 0.26) 3.11
Na1 2018 0.03 (-0.03, 0.09) 3.54
Na2 2018 0.17 (-0.00, 0.34) 2.47
Subtotal (I-squared = 69.4%, p = 0.000) 0.15 (0.09, 0.20) 38.35
Overall (I-squared = 83.7%, p = 0.000) 0.19 (0.15, 0.24) 100.00
NOTE: Weights are from random effects analysis
-1.15 0 1.15
C
Recurrence rate for surgery (subgrouped by surgery types)
Study ID | ES (95% CI) | % Weight
microscopic_surgery
Miguel 1982 0.07 (0.01, 0.13) 3.55
Schlechte 1985 0.32 (0.17, 0.47) 2.70
Giulio 1989 0.13 (0.03, 0.23) 3.17
Muriel1 2011 0.05 (-0.04, 0.13) 3.34
Muriel2 2011 0.20 (-0.05, 0.45) 1.81
Subtotal (I-squared = 65.7%, p = 0.020) 0.13 (0.05, 0.21) 14.57
mixed_surgery
Ronald1 1982 0.05 (-0.04, 0.14) 3.28
Ronald2 1982 0.07 (-0.06, 0.21) 2.86
Omar1 1983 0.50 (0.30, 0.70) 2.22
Omar2 1983 0.80 (0.45, 1.15) 1.19
Paul 1983 0.36 (0.17, 0.55) 2.33
Elise1 1984 0.14 (0.03, 0.25) 3.10
Elise2 1984 0.11 (-0.09, 0.31) 2.18
Fritz2 1985 0.91 (0.74, 1.08) 2.51
Charpentier 1985 0.17 (0.08, 0.26) 3.32
Fritz1 1985 0.31 (0.06, 0.56) 1.79
Jonathan 1992 0.08 (0.01, 0.15) 3.51
Fadi 1996 0.42 (0.29, 0.55) 2.95
Helen 1999 0.04 (-0.04, 0.12) 3.42
Kristof 2002 0.20 (-0.05, 0.45) 1.81
Marco 2002 0.17 (0.09, 0.25) 3.36
Esposito 2004 0.24 (0.06, 0.42) 2.38
Antonio 2007 0.14 (0.04, 0.24) 3.16
Kreutzer 2008 0.19 (0.11, 0.27) 3.39
Jae1 2009 0.05 (-0.02, 0.12) 3.47
Raverot 2010 0.32 (0.20, 0.44) 3.03
Xin 2011 0.20 (0.08, 0.32) 3.04
Vanessa 2012 0.34 (0.17, 0.51) 2.48
Hidetoshi 2013 0.06 (0.01, 0.11) 3.61
Diane1 2017 0.05 (-0.05, 0.15) 3.23
Diane2 2017 0.41 (0.18, 0.64) 1.92
Mario 2017 0.13 (-0.10, 0.35) 1.96
Na1 2018 0.03 (-0.03, 0.09) 3.54
Na2 2018 0.17 (-0.00, 0.34) 2.47
Subtotal (I-squared = 86.6%, p = 0.000) 0.21 (0.15, 0.27) 77.53
endoscopic_surgery
Jackson1 2010 0.14 (-0.12, 0.40) 1.73
Jackson2 2010 0.11 (-0.01, 0.22) 3.07
Yan-Long 2018 0.15 (0.04, 0.26) 3.11
Subtotal (I-squared = 0.0%, p = 0.865) 0.13 (0.05, 0.21) 7.90
Overall (I-squared = 83.7%, p = 0.000) 0.19 (0.15, 0.24) 100.00
NOTE: Weights are from random effects analysis
-1.15 0 1.15
D
Recurrence rate for DA (subgrouped by patients type)
Study ID | ES (95% CI) | % Weight
mixed_prolactinoma
Wang 1987 0.79 (0.63, 0.95) 5.13
Kharlip 2009 0.54 (0.40, 0.69) 5.30
Panagiotis 2011 0.42 (0.23, 0.61) 4.86
Sema 2016 0.46 (0.34, 0.58) 5.51
Margarida 2017 0.28 (0.16, 0.40) 5.47
Sandhya 2018 0.31 (0.08, 0.54) 4.49
Subtotal (I-squared = 81.6%, p = 0.000) 0.47 (0.32, 0.62) 30.76
macroprolactinoma
Johanna 1991 0.92 (0.76, 1.07) 5.19
Annamaria2 2007 0.53 (0.42, 0.64) 5.58
Thomas2 2011 0.93 (0.80, 1.06) 5.43
Ivan1 2015 0.23 (0.07, 0.39) 5.13
Paepegaey 2017 0.40 (0.24, 0.56) 5.13
Myoung 2017 0.58 (0.45, 0.71) 5.45
Subtotal (I-squared = 92.7%, p = 0.000) 0.60 (0.39, 0.81) 31.91
microprolactinoma
Muratori 1997 0.68 (0.47, 0.89) 4.66
Biswas 2005 0.64 (0.54, 0.74) 5.66
Annamaria1 2007 0.34 (0.25, 0.43) 5.75
Huda 2010 0.78 (0.65, 0.91) 5.43
Thomas1 2011 0.64 (0.50, 0.78) 5.33
Dogan 2015 0.81 (0.69, 0.93) 5.51
Myoung 2017 0.57 (0.39, 0.75) 4.99
Subtotal (I-squared = 89.2%, p = 0.000) 0.63 (0.49, 0.78) 37.33
Overall (I-squared = 89.2%, p = 0.000) 0.57 (0.48, 0.67) 100.00
NOTE: Weights are from random effects analysis
-1.07 0 1.07
E
Recurrence rate for DA (subgrouped by publication years)
Study ID | ES (95% CI) | % Weight
1980-1989
Wang 1987 0.79 (0.63, 0.95) 5.13
Subtotal (I-squared = .%, p = .) 0.79 (0.63, 0.95) 5.13
1990-1999
Johanna 1991 0.92 (0.76, 1.07) 5.19
Muratori 1997 0.68 (0.47, 0.89) 4.66
Subtotal (I-squared = 68.3%, p = 0.076) 0.81 (0.58, 1.04) 9.85
2000-2009
Annamaria1 2007 0.34 (0.25, 0.43) 5.75
Annamaria2 2007 0.53 (0.42, 0.64) 5.58
Kharlip 2009 0.54 (0.40, 0.69) 5.30
Biswas 2005 0.64 (0.54, 0.74) 5.66
Subtotal (I-squared = 85.9%, p = 0.000) 0.51 (0.37, 0.65) 22.28
2010-2019
Dogan 2015 0.81 (0.69, 0.93) 5.51
Paepegaey 2017 0.40 (0.24, 0.56) 5.13
Huda 2010 0.78 (0.65, 0.91) 5.43
Thomas1 2011 0.64 (0.50, 0.78) 5.33
Thomas2 2011 0.93 (0.80, 1.06) 5.43
Panagiotis 2011 0.42 (0.23, 0.61) 4.86
Ivan1 2015 0.23 (0.07, 0.39) 5.13
Sema 2016 0.46 (0.34, 0.58) 5.51
Myoung 2017 0.57 (0.39, 0.75) 4.99
Myoung 2017 0.58 (0.45, 0.71) 5.45
Margarida 2017 0.28 (0.16, 0.40) 5.47
Sandhya 2018 0.31 (0.08, 0.54) 4.49
Subtotal (I-squared = 90.0%, p = 0.000) 0.54 (0.41, 0.67) 62.74
Overall (I-squared = 89.2%, p = 0.000) 0.57 (0.48, 0.67) 100.00
NOTE: Weights are from random effects analysis
-1.07 0 1.07
F
Recurrence rate for DA (subgrouped by DAs types)
Study ID | ES (95% CI) | % Weight
bromocriptine
Wang 1987 0.79 (0.63, 0.95) 5.13
Johanna 1991 0.92 (0.76, 1.07) 5.19
Subtotal (I-squared = 15.5%, p = 0.277) 0.86 (0.73, 0.98) 10.32
cabergoline
Muratori 1997 0.68 (0.47, 0.89) 4.66
Annamaria1 2007 0.34 (0.25, 0.43) 5.75
Annamaria2 2007 0.53 (0.42, 0.64) 5.58
Kharlip 2009 0.54 (0.40, 0.69) 5.30
Dogan 2015 0.81 (0.69, 0.93) 5.51
Paepegaey 2017 0.40 (0.24, 0.56) 5.13
Subtotal (I-squared = 88.6%, p = 0.000) 0.55 (0.39, 0.70) 31.93
mixed_DA
Biswas 2005 0.64 (0.54, 0.74) 5.66
Huda 2010 0.78 (0.65, 0.91) 5.43
Thomas1 2011 0.64 (0.50, 0.78) 5.33
Thomas2 2011 0.93 (0.80, 1.06) 5.43
Panagiotis 2011 0.42 (0.23, 0.61) 4.86
Ivan1 2015 0.23 (0.07, 0.39) 5.13
Sema 2016 0.46 (0.34, 0.58) 5.51
Myoung 2017 0.57 (0.39, 0.75) 4.99
Myoung 2017 0.58 (0.45, 0.71) 5.45
Margarida 2017 0.28 (0.16, 0.40) 5.47
Sandhya 2018 0.31 (0.08, 0.54) 4.49
Subtotal (I-squared = 89.1%, p = 0.000) 0.54 (0.41, 0.66) 57.75
Overall (I-squared = 89.2%, p = 0.000) 0.57 (0.48, 0.67) 100.00
NOTE: Weights are from random effects analysis
-1.07 0 1.07
