# Supplementary material for: Are dopamine agonists still the first-choice treatment for prolactinoma in the era of endoscopy? A systematic review and meta-analysis
Source: Chin Neurosurg J. 2022 Apr 8;8:9. doi: 10.1186/s41016-022-00277-1 (PMC8994364; doi:10.1186/s41016-022-00277-1)
Supplement: Supplementary file 9 — Additional file 9: Supplementary Table 1. Basic characteristics of the included studies with surgery treatment. [file 41016_2022_277_MOESM9_ESM.docx]

Appendix table 1 Basic characteristics of the included studies with surgery treatment

| **Author** | **Year** | **Patient*** | **Intervention**** | **No.** | **Male/female** | **Mean age/y** | **Biochemical cure rate***** | **Recurrence rate****** | **Follow up duration******* |
| --- | --- | --- | --- | --- | --- | --- | --- | --- | --- |
| **Adam** | 2013 | mixed_p | endoscopic_s | 17 | NA | NA | 8/17 | NA | 40 |
| **Akira** | 2006 | mixed_p | mixed_s | 13 | 3/10 | NA | NA | NA | NA |
| **Alexander1** | 2018 | microp | endoscopic_s | 31 | 2/29 | 34 | 27/31 | NA | 32.4 |
| **Alexander2** | 2018 | microp | endoscopic_s | 29 | 8/21 | 33 | 13/29 | NA | 42 |
| **Amir1** | 2007 | microp | endoscopic_s | 12 | NA | NA | 11/12 | NA | NA |
| **Amir2** | 2007 | macrop | endoscopic_s | 13 | NA | NA | 10/13 | NA | NA |
| **Andreja1** | 2012 | microp | endoscopic_s | 39 | NA | NA | 38/39 | NA | NA |
| **Andreja2** | 2012 | macrop | endoscopic_s | 22 | NA | NA | 16/22 | NA | NA |
| **Antonio** | 2007 | mixed_p | mixed_s | 65 | 20/45 | 36 | 42/65 | 6/42 | 56 |
| **Arafah** | 1986 | mixed_p | microscopic_s | 120 | 0/120 | 27.9 | 96/120 | NA | NA |
| **Arimantas** | 2012 | microp | microscopic_s | 32 | 0/32 | 31 | 19/32 | NA | 50.4 |
| **Asano1** | 2001 | mixed_p | mixed_s | 8 | NA | 30.3 | 8/8 | NA | NA |
| **Berezin1** | 1995 | mixed_p | mixed_s | 23 | 23/0 | NA | NA | NA | NA |
| **Bevan** | 1987 | mixed_p | mixed_s | 67 | 19/48 | 32.4 | 34/67 | NA | NA |
| **Charpentier** | 1985 | mixed_p | mixed_s | 212 | NA | NA | 96/212 | 12/70 | 52.8 |
| **Der-Yang1** | 2002 | mixed_p | endoscopic_s | 22 | 0/22 | 45.3 | 15/22 | NA | NA |
| **Der-Yang2** | 2002 | mixed_p | microscopic_s | 22 | 1/21 | 46.7 | 17/22 | NA | NA |
| **Diane1** | 2017 | microp | mixed_s | 27 | NA | NA | 21/27 | 1/19 | 12 |
| **Diane2** | 2017 | macrop | mixed_s | 50 | NA | NA | 18/50 | 7/17 | 12 |
| **Elise1** | 1984 | microp | mixed_s | 42 | NA | NA | 37/42 | 5/37 | 50 |
| **Elise2** | 1984 | macrop | mixed_s | 23 | NA | NA | 9/23 | 1/9 | 50 |
| **Enrica** | 1989 | mixed_p | mixed_s | 22 | 1/21 | NA | NA | NA | NA |
| **Esposito** | 2004 | mixed_p | mixed_s | 42 | 14/26 | 33.2 | 25/42 | 5/21 | 31 |
| **Fadi** | 1996 | mixed_p | mixed_s | 64 | NA | NA | 59/64 | 25/59 | 147.6 |
| **Frederick1** | 2018 | mixed_p | endoscopic_s | 22 | 22/0 | 38 | 13/22 | NA | NA |
| **Frederick2** | 2018 | mixed_p | endoscopic_s | 57 | 0/57 | 35 | 52/57 | NA | NA |
| **Fritz1** | 1985 | microp | mixed_s | 13 | 0/13 | 28.7 | NA | 4/13 | NA |
| **Fritz2** | 1985 | macrop | mixed_s | 11 | 0/11 | 30.8 | NA | 10/11 | 26 |
| **Giorgio1** | 2006 | microp | endoscopic_s | 28 | NA | NA | 24/28 | NA | NA |
| **Giorgio2** | 2006 | macrop | endoscopic_s | 38 | NA | NA | 26/38 | NA | NA |
| **Giulio** | 1989 | mixed_p | mixed_s | 119 | 0/119 | NA | 73/119 | 5/40 | NA |
| **Hae-Dong** | 2001 | mixed_p | endoscopic_s | 35 | NA | NA | 24/35 | NA | NA |
| **Hae-Dong** | 1997 | mixed_p | endoscopic_s | 15 | 2/13 | 32.2 | 10/15 | NA | NA |
| **Hamilton** | 2005 | mixed_p | mixed_s | 79 | NA | NA | 34/79 | NA | NA |
| **Helen** | 1999 | microp | mixed_s | 32 | 0/32 | NA | 25/32 | 1/25 | 70 |
| **Hidemitsu** | 2001 | mixed_p | microscopic_s | 13 | NA | NA | NA | NA | NA |
| **Hidetoshi** | 2013 | mixed_p | mixed_s | 138 | NA | NA | 105/138 | 5/81 | 144 |
| **Hofstetter1** | 2011 | microp | endoscopic_s | 32 | NA | NA | 24/32 | NA | NA |
| **Hofstetter2** | 2011 | macrop | endoscopic_s | 53 | NA | NA | 27/53 | NA | NA |
| **Ivan2** | 2015 | microp | mixed_s | 40 | 23/17 | 43 | 18/40 | NA | NA |
| **Jackson1** | 2010 | microp | endoscopic_s | 7 | NA | NA | 6/7 | 0/7 | NA |
| **Jackson2** | 2010 | macrop | endoscopic_s | 34 | NA | NA | 28/34 | 3/28 | NA |
| **Jae1** | 2009 | mixed_p | mixed_s | 51 | 14/37 | 31.7 | 37/51 | 2/37 | NA |
| **Jae2** | 2009 | mixed_p | mixed_s | 19 | 2/17 | 46.6 | 19/19 | NA | NA |
| **Jonathan** | 1992 | mixed_p | mixed_s | 82 | 7/75 | 30.5 | 65/82 | 5/65 | 51.7 |
| **Kiyoshi** | 1984 | mixed_p | mixed_s | 12 | NA | NA | NA | NA | NA |
| **Kreutzer** | 2008 | mixed_p | mixed_s | 212 | 133/79 | 36 | 102/212 | 17/91 | NA |
| **Kristof** | 2002 | mixed_p | mixed_s | 37 | 16/21 | 31 | 10/37 | 2/10 | 44.4 |
| **Liang2** | 2018 | giant_p | mixed_s | 15 | NA | NA | 7/15 | NA | NA |
| **Lukas1** | 2017 | mixed_p | microscopic_s | 71 | 0/71 | 33.3 | 45/71 | NA | NA |
| **Marco** | 2002 | mixed_p | mixed_s | 120 | 27/93 | 29.7 | 77/120 | 13/77 | 50.2 |
| **Mario** | 2017 | microp | mixed_s | 24 | 5/19 | 34.8 | 8/24 | 1/8 | NA |
| **Michael** | 2009 | mixed_p | mixed_s | 176 | 20/156 | 31 | NA | NA | NA |
| **Miguel** | 1982 | mixed_p | microscopic_s | 100 | NA | NA | 68/100 | 5/68 | NA |
| **Muriel1** | 2011 | microp | microscopic_s | 24 | 4/20 | NA | 22/24 | 0/22 | 33.5 |
| **Muriel2** | 2011 | macrop | microscopic_s | 10 | 0/10 | NA | 10/10 | 2/10 | 33.5 |
| **Na1** | 2018 | microp | mixed_s | 31 | NA | 28 | 30/31 | 0/30 | 53 |
| **Na2** | 2018 | macrop | mixed_s | 32 | NA | 29 | 18/32 | 3/18 | 53 |
| **Naguib1** | 1986 | mixed_p | microscopic_s | 88 | 0/88 | 28.53 | 82/88 | NA | NA |
| **Oksana1** | 2018 | giant_p | mixed_s | 30 | 27/3 | 35.0 | 15/30 | NA | NA |
| **Omar1** | 1983 | microp | mixed_s | 28 | 0/28 | 25.9 | 24/28 | 12/24 | 48 |
| **Omar2** | 1983 | macrop | mixed_s | 16 | 0/16 | 28.4 | 5/16 | 4/5 | 30 |
| **Paluzzi1** | 2013 | microp | endoscopic_s | 11 | NA | NA | 10/11 | NA | NA |
| **Paluzzi2** | 2013 | macrop | endoscopic_s | 42 | NA | NA | 32/42 | NA | NA |
| **Paul** | 1983 | mixed_p | mixed_s | 40 | 0/40 | NA | 25/40 | 9/25 | 23 |
| **Pelkonen** | 1981 | mixed_p | mixed_s | 60 | 15/45 | NA | NA | NA | NA |
| **Pietro** | 2005 | mixed_p | mixed_s | 151 | NA | NA | 93/151 | NA | NA |
| **Raverot** | 2010 | mixed_p | mixed_s | 94 | 32/62 | 37.8 | 60/94 | 19/60 | 138 |
| **Ronald1** | 1982 | microp | mixed_s | 22 | NA | NA | NA | 1/21 | NA |
| **Ronald2** | 1982 | macrop | mixed_s | 14 | NA | NA | NA | 0/14 | NA |
| **Rudolf** | 1985 | microp | microscopic_s | 27 | NA | NA | 19/27 | NA | NA |
| **Safak1** | 2016 | macrop | endoscopic_s | 113 | NA | NA | 51/113 | NA | 36 |
| **Safak2** | 2016 | microp | endoscopic_s | 19 | NA | NA | 14/19 | NA | 36 |
| **Safak3** | 2016 | giant_p | endoscopic_s | 10 | NA | NA | 1/10 | NA | 36 |
| **Schlechte** | 1985 | mixed_p | microscopic_s | 68 | 0/68 | NA | 37/68 | 12/37 | 60.00 |
| **Shigetoshi1** | 2009 | microp | endoscopic_s | 17 | NA | NA | 16/17 | NA | NA |
| **Shigetoshi2** | 2009 | macrop | endoscopic_s | 12 | NA | NA | 5/12 | NA | NA |
| **Steven1** | 1996 | microp | mixed_s | 11 | 1/10 | 26 | 5/11 | NA | NA |
| **Steven2** | 1996 | macrop | mixed_s | 23 | 7/16 | 22 | 4/23 | NA | NA |
| **Taizo** | 1991 | mixed_p | mixed_s | 35 | 0/35 | NA | 22/35 | NA | NA |
| **Takakazu** | 2002 | mixed_p | mixed_s | 32 | 12/20 | 32 | 14/32 | NA | NA |
| **Thomson** | 1985 | mixed_p | microscopic_s | 77 | NA | NA | 53/77 | NA | NA |
| **Timothy** | 2015 | mixed_p | endoscopic_s | 66 | 22/44 | 36.7 | 45/66 | NA | 12 |
| **Vanessa** | 2012 | mixed_p | mixed_s | 63 | 18/45 | 31 | 29/63 | 10/29 | 36 |
| **Wang1** | 2015 | microp | endoscopic_s | 132 | NA | NA | 125/132 | NA | NA |
| **Wang2** | 2015 | macrop | endoscopic_s | 176 | NA | NA | 136/176 | NA | NA |
| **Winnie** | 2018 | mixed_p | mixed_s | 31 | 31/0 | 40.8 | NA | NA | 41.9 |
| **Wolfsberger** | 2003 | microp | mixed_s | 11 | 11/0 | 41 | 8/11 | NA | 84 |
| **Xin** | 2011 | mixed_p | mixed_s | 87 | 87/0 | 38 | 46/87 | 9/45 | 45 |
| **Yan** | 2015 | mixed_p | mixed_s | 99 | NA | NA | 71/99 | NA | NA |
| **Yang** | 2015 | mixed_p | mixed_s | 9 | 5/4 | NA | NA | NA | NA |
| **Yan-Long** | 2018 | mixed_p | endoscopic_s | 52 | 14/38 | 37.69 | 40/52 | 6/40 | 13.5 |
| **Yi** | 2018 | mixed_p | mixed_s | 36 | 11/25 | NA | 34/36 | NA | NA |
| **Yi-Jun** | 2017 | mixed_p | microscopic_s | 184 | 184/0 | 36.3 | 57/187 | NA | NA |
| **Youichi** | 1986 | mixed_p | microscopic_s | 98 | 16/82 | 31 | 45/98 | NA | NA |

* mixed_p: mixed_prolactinoma, data include patients with macroprolactinoma, microprolactinoma, and giant prolactinoma; macrop: macroprolactinoma; microp: microprolactinoma; giant_p: giant prolactinoma; ** mixed_s: mixed_surgery, data include patients with microscopic surgery and endoscopic surgery; microscopic_s: microscopic surgery; endoscopic_s: endoscopic surgery; *** cured/treated **** replased/cured ***** mean follow up duration months; NA：not applicable, because the data wasn’t provided by included studies.
