# Supplementary material for: Are dopamine agonists still the first-choice treatment for prolactinoma in the era of endoscopy? A systematic review and meta-analysis
Source: Chin Neurosurg J. 2022 Apr 8;8:9. doi: 10.1186/s41016-022-00277-1 (PMC8994364; doi:10.1186/s41016-022-00277-1)
Supplement: Supplementary file 10 — Additional file 10: Supplementary Table 2. Basic characteristics of the included studies with DAs treatment. [file 41016_2022_277_MOESM10_ESM.docx]

Appendix table 2 Basic characteristics of the included studies with DAs treatment

| **Author** | **Year** | **Patient*** | **Intervention**** | **No.** | **Male/female** | **Mean age/y** | **Biochemical cure rate***** | **Recurrence rate****** | **Follow up duration******* |
| --- | --- | --- | --- | --- | --- | --- | --- | --- | --- |
| **Albert** | 1992 | macrop | BRC | 29 | 14/15 | NA | NA | NA | NA |
| **Alessandro** | 2013 | mixed_p | CAB | 43 | 8/35 | 33.65 | 24/43 | NA | NA |
| **Amit** | 2015 | macrop | CAB | 71 | 71/0 | 44.7 | 51/71 | NA | 80.3 |
| **Annamaria1** | 2004 | mixed_p | CAB | 20 | 20/0 | 34 | 20/20 | NA | NA |
| **Annamaria1** | 2007 | microp | CAB | 115 | NA | NA | NA | 39/115 | 69 |
| **Annamaria2** | 2004 | macrop | CAB | 41 | 41/0 | 32.7 | 31/41 | NA | NA |
| **Annamaria2** | 2007 | macrop | CAB | 79 | NA | NA | NA | 42/79 | 68 |
| **Annamaria3** | 2004 | microp | CAB | 10 | 10/0 | 33.5 | 8/10 | NA | NA |
| **Annamaria5** | 1997 | microp | mixed_DAs | 8 | NA | NA | 8/8 | NA | NA |
| **Annamaria6** | 1997 | macrop | mixed_DAs | 19 | NA | NA | 15/19 | NA | NA |
| **Annamaria7** | 2000 | macrop | CAB | 26 | 11/15 | NA | 21/26 | NA | NA |
| **Annamaria8** | 2000 | macrop | mixed_DAs | 19 | 6/13 | NA | 18/19 | NA | NA |
| **Antonell1** | 2001 | macrop | BRC | 28 | NA | NA | 13/28 | NA | 12 |
| **Antonell2** | 2001 | microp | BRC | 44 | NA | NA | 25/44 | NA | 12 |
| **Antonell3** | 2001 | macrop | CAB | 56 | NA | NA | 46/56 | NA | 6 |
| **Antonell4** | 2001 | microp | CAB | 60 | NA | NA | 54/60 | NA | 6 |
| **Archer** | 1982 | microp | BRC | 17 | 0/17 | NA | 16/17 | NA | 24 |
| **Arijit1** | 2005 | macrop | BRC | 15 | 15/0 | 30 | NA | NA | NA |
| **Arijit2** | 2005 | giant_p | BRC | 14 | 14/0 | 34 | NA | NA | NA |
| **Arturo** | 1979 | mixed_p | BRC | 14 | 0/14 | 29.71 | 10/14 | NA | NA |
| **Asano2** | 2001 | mixed_p | BRC | 5 | NA | 48.6 | NA | NA | NA |
| **Ashu1** | 2013 | macrop | CAB | 19 | 10/9 | 37.4 | 15/19 | NA | 15.5 |
| **Ashu1** | 2012 | macrop | CAB | 19 | NA | NA | 14/19 | NA | 6 |
| **Ashu2** | 2013 | macrop | CAB | 19 | 11/8 | 31 | 18/19 | NA | 16.6 |
| **Ashu2** | 2012 | macrop | CAB | 19 | NA | NA | 16/19 | NA | 6 |
| **Barbara** | 2017 | mixed_p | BRC | 28 | 0/28 | 26 | 13/28 | NA | NA |
| **Barbosa** | 2014 | mixed_p | mixed_DAs | 21 | NA | NA | 17/21 | NA | NA |
| **Berezin2** | 1995 | mixed_p | mixed_DAs | 52 | 52/0 | NA | 36/52 | NA | NA |
| **Bhansali** | 2010 | macrop | CAB | 15 | NA | 31.7 | 14/15 | NA | NA |
| **Biswas** | 2005 | microp | mixed_DAs | 89 | NA | NA | NA | 57/89 | 37.2 |
| **Cannavo1** | 1999 | microp | CAB | 26 | 4/22 | NA | 23/26 | NA | NA |
| **Cannavo2** | 1999 | macrop | CAB | 11 | 1/10 | NA | 11/11 | NA | NA |
| **Carlo** | 1992 | mixed_p | CAB | 127 | 3/124 | NA | 114/127 | NA | NA |
| **Catarina** | 2018 | macrop | mixed_DAs | 67 | 34/33 | 43 | 58/67 | NA | NA |
| **Christine1** | 2016 | microp | mixed_DAs | 27 | 7/20 | 30.1 | NA | NA | NA |
| **Christine2** | 2016 | macrop | mixed_DAs | 30 | 23/7 | 44.2 | NA | NA | NA |
| **Cintia** | 2011 | mixed_p | mixed_DAs | 22 | NA | NA | 17/22 | NA | NA |
| **Coculescu** | 1983 | mixed_p | BRC | 22 | NA | NA | 19/22 | NA | NA |
| **Corsello** | 2003 | giant_p | CAB | 10 | NA | NA | 5/10 | NA | NA |
| **Dogan** | 2015 | microp | CAB | 42 | NA | NA | NA | 34/42 | 12 |
| **Emir** | 2018 | mixed_p | mixed_DAs | 25 | 18/7 | 39.96 | NA | NA | NA |
| **Erika1** | 2007 | mixed_p | mixed_DAs | 31 | 0/31 | 33.0 | NA | NA | NA |
| **Erika2** | 2007 | mixed_p | mixed_DAs | 45 | 0/45 | 34.5 | NA | NA | NA |
| **Essais** | 2002 | macrop | BRC | 29 | 10/19 | NA | 27/29 | NA | NA |
| **Etienne** | 1996 | mixed_p | mixed_DAs | 10 | 2/8 | NA | 8/9 | NA | NA |
| **Etienne** | 2009 | macrop | CAB | 122 | 50/72 | NA | 115/122 | NA | NA |
| **Etual1** | 2016 | giant_p | mixed_DAs | 47 | 42/5 | 44 | 32/47 | NA | NA |
| **Etual2** | 2016 | macrop | mixed_DAs | 152 | 72/80 | 40 | 113/152 | NA | NA |
| **Eun-Hee** | 2009 | macrop | CAB | 10 | 10/0 | 37 | 6/10 | NA | NA |
| **Ferrari** | 1997 | macrop | CAB | 85 | NA | NA | 52/85 | NA | NA |
| **Hancock** | 1980 | mixed_p | BRC | 36 | NA | NA | 28/36 | NA | NA |
| **Hildebrandt** | 1989 | macrop | BRC | 10 | NA | NA | 3/10 | NA | NA |
| **Hildebrandt** | 1992 | mixed_p | mixed_DAs | 14 | NA | NA | 10/14 | NA | NA |
| **Huda** | 2010 | microp | mixed_DAs | 40 | 1/39 | NA | NA | 31/40 | 58 |
| **Ilan** | 2007 | giant_p | CAB | 10 | 10/0 | 38.2 | 9/10 | NA | NA |
| **Ilan** | 2016 | giant_p | mixed_DAs | 18 | 16/2 | 36.3 | 11/18 | NA | NA |
| **Ilan** | 2019 | mixed_p | mixed_DAs | 28 | 28/0 | 71.3 | 24/27 | NA | NA |
| **Ivan1** | 2015 | macrop | mixed_DAs | 38 | 0/38 | 36.5 | 26/38 | 6/26 | NA |
| **Jae3** | 2009 | mixed_p | mixed_DAs | 47 | 15/32 | 34.1 | 47/47 | NA | NA |
| **Johanna** | 1991 | macrop | BRC | 12 | 8/4 | 42.2 | NA | 11/12 | 12 |
| **Johanna** | 1990 | macrop | BRC | 19 | 12/7 | NA | 16/19 | NA | 40.8 |
| **Katarina** | 2011 | mixed_p | mixed_DAs | 14 | 6/8 | 39.7 | 14/14 | NA | NA |
| **Kharlip** | 2009 | mixed_p | CAB | 46 | NA | NA | NA | 25/46 | NA |
| **Kyung** | 2013 | mixed_p | BRC | 23 | 17/6 | 48 | 16/23 | NA | NA |
| **Liang1** | 2018 | giant_p | mixed_DAs | 27 | NA | NA | 14/27 | NA | NA |
| **Lukas2** | 2017 | mixed_p | mixed_DAs | 36 | 0/36 | 35.5 | 20/36 | NA | NA |
| **Margarida** | 2017 | mixed_p | mixed_DAs | 50 | 5/45 | 35.1 | NA | 14/50 | NA |
| **Maria** | 2015 | mixed_p | mixed_DAs | 29 | NA | NA | 29/29 | NA | NA |
| **María1 Martín** | 2013 | microp | mixed_DAs | 20 | NA | 32.8 | 12/20 | NA | NA |
| **María2 Martín** | 2013 | microp | mixed_DAs | 27 | NA | 27.9 | 27/27 | NA | NA |
| **Masami** | 2010 | mixed_p | CAB | 85 | NA | NA | 85/85 | NA | NA |
| **Mia-Maiken** | 2013 | mixed_p | mixed_DAs | 12 | 5/7 | 39.7 | 8/12 | NA | NA |
| **Moon** | 2011 | mixed_p | BRC | 36 | 25/11 | NA | 29/36 | NA | NA |
| **Muratori** | 1997 | microp | CAB | 26 | 0/26 | NA | 25/26 | 13/19 | 12 |
| **Mussa** | 2015 | giant_p | CAB | 16 | 10/6 | 34.9 | 6/16 | NA | NA |
| **Myoung1** | 2017 | microp | mixed_DAs | 30 | 1/29 | 33 | NA | 17/30 | 26 |
| **Myoung2** | 2017 | macrop | mixed_DAs | 59 | 26/33 | 34 | NA | 34/59 | 25.7 |
| **Naguib1** | 1986 | mixed_p | BRC | 102 | 0/102 | 28.7 | NA | NA | NA |
| **Nazir** | 2015 | mixed_p | CAB | 19 | 1/18 | 27.3 | 18/19 | NA | NA |
| **Niki** | 2013 | macrop | CAB | 12 | 11/1 | 40.5 | 11/12 | NA | NA |
| **Nissim** | 1982 | macrop | BRC | 7 | NA | NA | 4/7 | NA | NA |
| **Oksana2** | 2018 | giant_p | mixed_DAs | 38 | 33/5 | 46.6 | 20/38 | NA | NA |
| **Oluwaseun** | 2019 | mixed_p | mixed_DAs | 69 | NA | NA | 29/69 | NA | 6 |
| **Paepegaey** | 2017 | macrop | CAB | 260 | 135/125 | 36.2 | 157/260 | 14/35 | NA |
| **Panagiotis** | 2011 | mixed_p | mixed_DAs | 79 | 17/62 | 35.3 | NA | 11/26 | 49 |
| **Renata** | 2013 | mixed_p | CAB | 61 | 13/48 | 34.4 | 57/61 | NA | 60 |
| **Renata** | 2015 | mixed_p | CAB | 32 | 32/0 | 42 | 31/32 | NA | 24 |
| **Sandhya** | 2018 | mixed_p | mixed_DAs | 28 | 0/28 | NA | 16/18 | 5/16 | 12 |
| **Sandhya** | 2017 | mixed_p | mixed_DAs | 16 | 0/16 | NA | 15/16 | NA | NA |
| **Sema** | 2016 | mixed_p | mixed_DAs | 67 | 17/50 | NA | NA | 31/67 | 108.8 |
| **Sema1** | 2018 | mixed_p | mixed_DAs | 257 | NA | 36 | NA | NA | NA |
| **Sema2** | 2018 | mixed_p | mixed_DAs | 51 | NA | 35 | NA | NA | NA |
| **Shrikrishna** | 2009 | mixed_p | mixed_DAs | 39 | 9/30 | NA | 14/39 | NA | NA |
| **Shrikrishna** | 2010 | giant_p | CAB | 10 | 5/5 | 36.1 | 8/10 | NA | NA |
| **Tevfik1** | 2001 | mixed_p | BRC | 17 | 2/15 | 33.4 | 10/17 | NA | NA |
| **Tevfik2** | 2001 | mixed_p | CAB | 17 | 2/15 | 32.7 | 14/17 | NA | NA |
| **Thomas1** | 2011 | microp | mixed_DAs | 45 | NA | NA | NA | 29/45 | 4.8 |
| **Thomas2** | 2011 | macrop | mixed_DAs | 15 | NA | NA | NA | 14/15 | 8.8 |
| **Verena** | 2017 | mixed_p | CAB | 53 | 31/22 | 40 | NA | NA | NA |
| **Wang** | 1987 | mixed_p | BRC | 24 | NA | NA | NA | 19/24 | 40.8 |
| **Youngki** | 2014 | macrop | mixed_DAs | 44 | 28/16 | 36.8 | 34/44 | NA | NA |

* mixed_p: mixed_prolactinoma, data include patients with macroprolactinoma, microprolactinoma, and giant prolactinoma; macrop: macroprolactinoma; microp: microprolactinoma; giant_p: giant prolactinoma; ** mixed_DAs: data include patients using bromocriptine and cabergoline; CAB: cabergoline; BRC: bromocriptine; *** cured/treated **** replased/cured ***** mean follow up duration months; NA: not applicable, because the data wasn’t provided by included studies.
