# Supplementary material for: Are dopamine agonists still the first-choice treatment for prolactinoma in the era of endoscopy? A systematic review and meta-analysis
Source: Chin Neurosurg J. 2022 Apr 8;8:9. doi: 10.1186/s41016-022-00277-1 (PMC8994364; doi:10.1186/s41016-022-00277-1)
Supplement: Supplementary file 11 — Additional file 11: Supplementary Table 3. Summary table of risk of bias for RCT. [file 41016_2022_277_MOESM11_ESM.docx]

Appendix table 3 Summary of risk of bias assessment for randomized controlled trials.

| **Study** | **Randomisation process** | **Deviations from intended interventions** | **Missing outcome data** | **Measurement of the outcome** | **Selection of the reported result** | **Overall bias** |
| --- | --- | --- | --- | --- | --- | --- |
| **Ashu 2013** | Low | Low | Low | Low | Some concerns | Some concerns |
| **Ashu 2012** | Some concerns | Low | Low | Low | Some concerns | Some concerns |
| **Der-Yang 2002** | Some concerns | High | Low | Low | Some concerns | High |
| **Tevfik 2001** | Some concerns | Low | Low | Low | Some concerns | Some concerns |
