# Supplementary material for: Are dopamine agonists still the first-choice treatment for prolactinoma in the era of endoscopy? A systematic review and meta-analysis
Source: Chin Neurosurg J. 2022 Apr 8;8:9. doi: 10.1186/s41016-022-00277-1 (PMC8994364; doi:10.1186/s41016-022-00277-1)
Supplement: Supplementary file 12 — Additional file 12: Supplementary Table 4. Summary table of risk of bias for non-RCT. [file 41016_2022_277_MOESM12_ESM.docx]

Appendix table 4 Summary of risk of bias assessment for non-randomized controlled trials.

| **Study** | **D1** | **D2** | **D3** | **D4** | **D5** | **D6** | **D7** | **Overall bias** |
| --- | --- | --- | --- | --- | --- | --- | --- | --- |
| **Albert 1992** | Serious | Low | Low | Low | Low | Low | Low | Serious |
| **Alexander 2018** | Low | Low | Low | Low | Low | Low | Low | Low |
| **Annamaria1 2004** | Low | Moderate | Low | Low | Low | Low | Low | Moderate |
| **Annamaria2 2004** | Low | Low | Low | Low | Low | Low | Low | Low |
| **Annamaria 2000** | Serious | Low | Low | Low | Moderate | Low | Low | Serious |
| **Antonell 2001** | Serious | Low | Low | Low | Low | Low | Low | Serious |
| **Asano 2001** | Low | Low | Low | Low | Low | Low | Low | Low |
| **Barbosa 2014** | Low | Low | Low | Low | Low | Low | Low | Low |
| **Biswas 2005** | Moderate | Low | Low | Low | Low | Low | Low | Moderate |
| **Christine 2016** | Low | Low | Low | Low | Low | Low | Low | Low |
| **Cintia 2011** | Low | Low | Low | Low | Low | Low | Low | Low |
| **Dogan 2015** | Low | Low | Low | Low | Low | Low | Low | Low |
| **Emir 2018** | Low | Low | Low | Low | Moderate | Moderate | Low | Moderate |
| **Erika1 2007** | Low | Low | Low | Low | Low | Low | Low | Low |
| **Erika2 2007** | Low | Low | Low | Low | Low | Low | Low | Low |
| **Etienne 1996** | Serious | Low | Low | Low | Low | Low | Low | Serious |
| **Etual 2016** | Low | Low | Low | Low | Low | Low | Low | Low |
| **Frederick 2018** | Low | Low | Low | Low | Low | Low | Low | Low |
| **Hamilton 2005** | Low | Low | Low | Low | Low | Low | Low | Low |
| **Hildebrandt 1992** | Low | Low | Low | Serious | Serious | Low | Serious | Serious |
| **Ivan 2015** | Low | Low | Moderate | Low | Low | Low | Low | Moderate |
| **Kreutzer 2008** | Serious | Low | Low | Low | Moderate | Low | Low | Serious |
| **Liang 2018** | Low | Low | Low | Low | Low | Low | Low | Low |
| **Lukas 2017** | Low | Low | Low | Low | Low | Low | Low | Low |
| **Margarida 2017** | Low | Low | Low | Low | Low | Low | Low | Low |
| **Mario 2017** | Low | Moderate | Low | Low | Low | Low | Moderate | Moderate |
| **Michael 2009** | Low | Low | Low | Low | Low | Low | Low | Low |
| **Naguib 1986** | Low | Low | Low | Low | Low | Low | Low | Low |
| **Nazir 2015** | Low | Low | Low | Low | Moderate | Low | Low | Moderate |
| **Renata 2015** | Low | Low | Low | Low | Low | Low | Low | Low |
| **Sema 2016** | Low | Low | Low | Low | Low | Low | Low | Low |
| **Sema 2018** | Low | Low | Low | Low | Low | Moderate | Low | Moderate |

D1: Bias due to confounding

D2: Bias in selection of participants into the study

D3: Bias in classification of interventions

D4: Bias due to deviations from intended interventions

D5: Bias due to missing data

D6: Bias in measurement of outcomes

D7: Bias in selection of the reported result
