# Supplementary material for: Are dopamine agonists still the first-choice treatment for prolactinoma in the era of endoscopy? A systematic review and meta-analysis
Source: Chin Neurosurg J. 2022 Apr 8;8:9. doi: 10.1186/s41016-022-00277-1 (PMC8994364; doi:10.1186/s41016-022-00277-1)
Supplement: Supplementary file 13 — Additional file 13: Supplementary Table 5. Summary table of risk of bias for case-series study. [file 41016_2022_277_MOESM13_ESM.docx]

Appendix table 5 Summary of risk of bias assessment for case-series studies.

| **Study** | **Q1** | **Q2** | **Q3** | **Q4** | **Q5** | **Q6** | **Q7** | **Q8** | **Q9** | **Q10** | **Q11** | **Q12** | **Q13** | **Q14** | **Q15** | **Q16** | **Q17** | **Q18** | **Q19** | **Q20** | **Score** |
| --- | --- | --- | --- | --- | --- | --- | --- | --- | --- | --- | --- | --- | --- | --- | --- | --- | --- | --- | --- | --- | --- |
| **Adam 2013** | Y | N | N | Y | Y | Y | U | Y | N | Y | U | Y | Y | U | Y | N | Y | Y | Y | Y | 13 |
| **Akira 2006** | Y | N | N | Y | Y | Y | U | Y | N | Y | U | Y | U | Y | U | N | Y | Y | Y | Y | 12 |
| **Alessandro 2013** | Y | N | N | Y | Y | Y | U | Y | N | Y | U | Y | Y | Y | Y | N | Y | Y | Y | Y | 14 |
| **Amir 2007** | Y | N | N | Y | Y | Y | U | Y | N | Y | U | Y | Y | U | Y | N | Y | Y | Y | N | 12 |
| **Amit 2015** | Y | Y | N | Y | Y | Y | U | Y | N | Y | U | Y | Y | Y | Y | N | Y | Y | Y | Y | 15 |
| **Andreja 2012** | Y | Y | N | Y | Y | Y | U | Y | N | Y | U | Y | Y | Y | Y | N | Y | Y | Y | Y | 15 |
| **Annamaria 2007** | Y | Y | U | U | Y | Y | U | Y | N | Y | U | Y | Y | Y | Y | Y | Y | Y | Y | Y | 15 |
| **Annamaria 1997** | Y | U | U | U | Y | Y | U | Y | N | Y | U | Y | Y | Y | Y | N | Y | Y | Y | N | 12 |
| **Antonio 2007** | Y | U | Y | Y | Y | Y | U | Y | N | Y | U | Y | Y | Y | Y | N | N | Y | Y | N | 13 |
| **Arafah 1986** | Y | U | U | U | Y | Y | U | Y | N | Y | U | Y | Y | U | Y | N | Y | Y | Y | N | 11 |
| **Archer 1982** | Y | U | U | U | Y | Y | U | Y | N | Y | U | Y | Y | U | U | N | N | Y | Y | N | 9 |
| **Arijit 2005** | Y | U | N | Y | Y | Y | Y | Y | N | Y | U | Y | Y | Y | Y | N | Y | Y | Y | N | 14 |
| **Arimantas 2012** | Y | Y | N | Y | Y | Y | Y | Y | N | Y | U | Y | Y | Y | Y | N | Y | Y | Y | Y | 16 |
| **Arturo 1979** | U | U | U | U | Y | U | U | Y | N | Y | U | Y | Y | U | Y | N | Y | Y | Y | Y | 10 |
| **Barbara 2017** | Y | N | N | U | Y | Y | U | Y | N | Y | U | Y | Y | Y | Y | N | Y | Y | Y | Y | 13 |
| **Berezin 1995** | Y | N | N | U | Y | U | U | Y | N | Y | U | Y | Y | U | Y | N | Y | Y | Y | N | 10 |
| **Bevan 1987** | U | U | U | U | Y | Y | U | Y | N | Y | U | Y | Y | U | Y | N | Y | Y | Y | N | 10 |
| **Bhansali 2010** | Y | Y | N | Y | Y | Y | U | Y | N | Y | U | Y | Y | Y | Y | N | Y | Y | Y | Y | 15 |
| **Cannavo 1999** | Y | U | U | U | Y | Y | U | Y | N | Y | U | Y | Y | Y | U | N | Y | Y | Y | N | 11 |
| **Carlo 1992** | N | U | U | U | Y | Y | U | Y | N | Y | U | Y | Y | U | Y | N | Y | Y | Y | N | 10 |
| **Catarina 2018** | Y | N | Y | U | Y | Y | U | Y | N | Y | U | Y | Y | U | Y | N | Y | Y | Y | Y | 13 |
| **Charpentier 1985** | Y | N | Y | U | Y | Y | U | Y | N | Y | U | Y | Y | Y | Y | N | Y | Y | Y | N | 13 |
| **Coculescu 1983** | N | U | U | U | U | Y | U | Y | N | N | U | U | U | U | Y | U | U | U | Y | U | 4 |
| **Corsello 2003** | Y | Y | N | U | Y | Y | U | Y | N | Y | U | Y | Y | Y | Y | N | Y | Y | Y | N | 13 |
| **Diane 2017** | Y | N | N | U | Y | Y | U | Y | N | Y | U | Y | Y | Y | Y | N | Y | Y | Y | Y | 13 |
| **Elise 1984** | U | U | N | U | Y | Y | U | Y | N | Y | U | Y | Y | Y | Y | N | Y | Y | Y | Y | 12 |
| **Enrica 1989** | U | U | N | U | Y | Y | U | Y | N | Y | U | Y | Y | U | Y | N | Y | Y | Y | Y | 11 |
| **Esposito 2004** | Y | U | N | Y | Y | Y | U | Y | N | Y | U | Y | Y | Y | Y | N | Y | Y | Y | N | 13 |
| **Essais 2002** | Y | U | Y | U | Y | Y | U | Y | N | Y | U | U | U | U | Y | U | U | U | Y | U | 8 |
| **Etienne 2009** | Y | N | N | U | Y | Y | U | Y | N | Y | U | Y | Y | Y | Y | N | Y | Y | Y | Y | 13 |
| **Eun-Hee 2009** | Y | N | N | U | Y | Y | U | Y | N | Y | U | Y | Y | Y | Y | N | Y | Y | Y | Y | 13 |
| **Fadi 1996** | Y | N | N | U | Y | Y | U | Y | N | Y | U | Y | Y | Y | Y | N | Y | Y | Y | N | 12 |
| **Ferrari 1997** | Y | U | Y | U | Y | Y | U | Y | N | Y | U | Y | Y | U | U | N | Y | Y | Y | N | 11 |
| **Fritz 1985** | Y | U | U | U | Y | Y | U | Y | N | Y | U | Y | Y | Y | Y | N | Y | Y | Y | N | 12 |
| **Giorgio 2006** | Y | U | N | U | Y | Y | U | Y | N | Y | U | Y | Y | U | Y | N | N | Y | Y | N | 10 |
| **Giulio 1989** | Y | U | U | Y | Y | Y | U | Y | N | Y | U | Y | Y | U | U | N | N | Y | Y | N | 10 |
| **Hae-Dong 2001** | Y | N | N | U | Y | Y | U | Y | N | Y | U | Y | Y | U | U | N | N | Y | Y | N | 9 |
| **Hae-Dong 1997** | Y | N | N | U | Y | Y | U | Y | N | Y | U | Y | Y | U | U | N | N | Y | Y | N | 9 |
| **Hancock 1980** | Y | U | U | U | Y | Y | U | Y | N | Y | U | Y | Y | U | U | N | N | Y | Y | N | 9 |
| **Helen 1999** | Y | N | N | U | Y | Y | U | Y | N | Y | U | Y | Y | Y | Y | N | N | Y | Y | N | 11 |
| **Hidemitsu 2001** | Y | U | U | U | Y | Y | U | Y | N | Y | U | Y | Y | U | U | N | N | Y | Y | N | 9 |
| **Hidetoshi 2013** | Y | N | N | U | N | Y | U | Y | N | Y | U | Y | Y | U | Y | N | N | Y | Y | N | 9 |
| **Hildebrandt 1989** | U | U | N | U | N | Y | U | Y | N | Y | U | Y | Y | U | U | N | N | Y | Y | N | 7 |
| **Hofstetter 2011** | Y | N | N | Y | Y | Y | U | Y | N | Y | U | Y | Y | U | Y | N | Y | Y | Y | Y | 13 |
| **Huda 2010** | Y | U | N | U | Y | Y | U | Y | N | Y | U | Y | Y | Y | Y | N | Y | Y | Y | Y | 13 |
| **Ilan 2007** | Y | N | Y | U | Y | Y | U | Y | N | Y | U | Y | Y | Y | U | N | Y | Y | Y | N | 12 |
| **Ilan 2016** | Y | N | Y | U | Y | Y | U | Y | N | Y | U | Y | Y | Y | Y | N | Y | Y | Y | Y | 14 |
| **Ilan 2019** | Y | U | Y | U | Y | Y | U | Y | N | Y | U | Y | Y | Y | Y | N | Y | Y | Y | Y | 14 |
| **Jackson 2010** | Y | N | N | Y | Y | Y | U | Y | N | Y | U | Y | Y | U | U | N | Y | Y | Y | N | 11 |
| **Jae 2009** | Y | N | N | U | Y | Y | U | Y | N | Y | U | Y | Y | U | U | N | Y | Y | Y | Y | 11 |
| **Johanna 1991** | Y | U | N | U | Y | Y | U | Y | N | Y | U | Y | Y | U | Y | N | Y | Y | Y | N | 11 |
| **Johanna 1990** | Y | U | N | Y | N | Y | U | Y | N | Y | U | Y | Y | U | Y | N | Y | Y | Y | N | 11 |
| **Jonathan 1992** | Y | N | N | U | N | Y | U | Y | N | Y | U | Y | Y | U | Y | N | Y | Y | Y | N | 10 |
| **Katarina 2011** | Y | U | N | Y | Y | Y | U | Y | N | Y | U | Y | Y | Y | U | N | Y | Y | Y | Y | 13 |
| **Kharlip 2009** | Y | N | N | U | N | Y | U | Y | N | Y | U | Y | Y | U | Y | N | N | Y | Y | N | 9 |
| **Kiyoshi 1984** | Y | U | U | U | Y | Y | U | Y | N | Y | U | Y | Y | U | U | N | Y | Y | Y | N | 10 |
| **Kristof 2002** | Y | N | U | Y | Y | Y | U | Y | N | Y | U | Y | Y | Y | Y | N | Y | Y | Y | N | 13 |
| **Kyung 2013** | Y | U | N | U | Y | Y | U | Y | N | Y | U | Y | Y | Y | U | N | Y | Y | Y | Y | 12 |
| **Marco 2002** | Y | U | N | Y | Y | Y | U | Y | N | Y | U | Y | Y | Y | Y | N | Y | Y | Y | N | 13 |
| **Maria 2015** | Y | N | U | U | Y | Y | U | Y | N | Y | U | Y | Y | Y | Y | Y | Y | Y | Y | Y | 14 |
| **María Martín 2013** | Y | N | N | U | Y | Y | U | Y | N | Y | U | Y | Y | Y | Y | N | N | Y | Y | Y | 12 |
| **Masami 2010** | Y | U | U | U | Y | Y | U | Y | N | N | U | U | U | U | Y | U | U | Y | Y | N | 7 |
| **Mia-Maiken 2013** | Y | U | N | Y | Y | Y | U | Y | N | Y | U | Y | Y | Y | Y | N | Y | Y | Y | Y | 14 |
| **Miguel 1982** | Y | U | N | Y | Y | Y | U | Y | N | Y | U | Y | Y | U | Y | N | Y | Y | Y | Y | 13 |
| **Moon 2011** | Y | N | N | U | Y | Y | U | Y | N | Y | U | Y | Y | N | Y | Y | Y | Y | Y | Y | 13 |
| **Muratori 1997** | Y | U | U | U | Y | Y | U | Y | N | Y | U | Y | Y | Y | Y | Y | Y | Y | Y | Y | 14 |
| **Muriel 2011** | Y | N | N | Y | Y | Y | U | Y | N | Y | U | Y | Y | Y | Y | N | Y | Y | Y | Y | 14 |
| **Mussa 2015** | Y | N | N | U | Y | Y | U | Y | N | Y | U | Y | Y | Y | Y | N | Y | Y | Y | Y | 13 |
| **Myoung 2017** | Y | N | N | U | Y | Y | U | Y | N | Y | U | Y | Y | Y | Y | Y | Y | Y | Y | Y | 14 |
| **Na 2018** | Y | U | N | Y | Y | Y | U | Y | N | Y | U | Y | Y | Y | Y | N | Y | Y | Y | Y | 14 |
| **Niki 2013** | Y | Y | N | U | Y | Y | U | Y | N | Y | U | Y | Y | U | Y | Y | Y | Y | Y | N | 13 |
| **Nissim 1982** | Y | U | U | U | Y | Y | U | Y | N | Y | U | Y | Y | U | U | N | Y | Y | Y | Y | 11 |
| **Oksana 2018** | Y | N | N | U | Y | Y | U | Y | N | Y | U | Y | Y | Y | Y | N | Y | Y | Y | Y | 13 |
| **Oluwaseun 2019** | Y | N | Y | U | Y | Y | U | Y | N | Y | U | Y | Y | Y | Y | N | Y | Y | Y | Y | 14 |
| **Omar 1983** | Y | U | N | U | Y | Y | U | Y | N | Y | U | Y | Y | Y | Y | N | Y | Y | Y | Y | 13 |
| **Paepegaey 2017** | Y | N | N | U | Y | Y | U | Y | N | Y | U | Y | Y | Y | Y | N | Y | Y | Y | Y | 13 |
| **Paluzzi 2013** | Y | N | N | U | Y | Y | U | Y | N | Y | U | Y | Y | U | Y | N | Y | Y | Y | Y | 12 |
| **Panagiotis 2011** | Y | N | N | U | Y | Y | U | Y | N | Y | U | Y | Y | Y | Y | N | Y | Y | Y | Y | 13 |
| **Paul 1983** | Y | N | U | U | Y | Y | U | Y | N | Y | U | Y | Y | Y | Y | N | Y | Y | Y | Y | 13 |
| **Pelkonen 1981** | Y | U | U | U | Y | Y | U | Y | N | Y | U | Y | Y | U | Y | N | Y | Y | Y | N | 11 |
| **Pietro 2005** | Y | Y | U | Y | Y | Y | U | Y | N | Y | U | Y | Y | U | Y | N | Y | Y | Y | N | 13 |
| **Raverot 2010** | Y | N | U | U | Y | Y | U | Y | N | Y | U | Y | Y | U | Y | N | Y | Y | Y | Y | 12 |
| **Renata 2013** | Y | U | N | Y | Y | Y | U | Y | N | Y | U | Y | Y | U | Y | N | Y | Y | Y | N | 12 |
| **Ronald 1982** | Y | U | U | Y | Y | Y | U | Y | N | Y | U | Y | Y | U | Y | N | Y | Y | Y | N | 12 |
| **Rudolf 1985** | U | U | N | U | N | U | U | Y | N | U | U | Y | U | U | Y | N | Y | Y | Y | N | 6 |
| **Safak 2016** | Y | N | U | Y | N | Y | U | Y | N | Y | U | Y | Y | Y | Y | N | Y | Y | Y | Y | 13 |
| **Sandhya 2018** | Y | N | N | U | Y | Y | U | Y | N | Y | U | Y | Y | Y | Y | N | Y | Y | Y | Y | 13 |
| **Sandhya 2017** | Y | N | N | U | Y | Y | U | Y | N | Y | U | Y | Y | Y | Y | N | Y | Y | Y | Y | 13 |
| **Schlechte 1985** | Y | U | N | U | Y | Y | U | Y | N | Y | U | Y | Y | Y | U | N | Y | Y | Y | N | 11 |
| **Shigetoshi 2009** | Y | U | Y | U | Y | Y | U | Y | N | Y | U | Y | Y | U | U | N | Y | Y | Y | N | 11 |
| **Shrikrishna 2009** | U | N | U | U | Y | Y | U | Y | N | Y | U | Y | Y | U | Y | Y | Y | Y | Y | N | 11 |
| **Shrikrishna 2010** | Y | U | N | U | Y | Y | U | Y | N | Y | U | Y | Y | Y | U | N | Y | Y | Y | Y | 12 |
| **Steven 1996** | Y | N | N | U | Y | Y | U | Y | N | Y | U | Y | Y | Y | Y | N | Y | Y | Y | N | 12 |
| **Taizo 1991** | Y | N | N | U | Y | Y | U | Y | N | Y | U | Y | Y | Y | Y | N | Y | Y | Y | N | 12 |
| **Takakazu 2002** | Y | U | N | U | Y | Y | U | Y | N | Y | U | Y | Y | Y | U | N | Y | Y | Y | N | 11 |
| **Thomas 2011** | Y | N | N | U | Y | Y | U | Y | N | Y | U | Y | Y | Y | U | N | Y | Y | Y | Y | 12 |
| **Thomson 1985** | Y | N | U | U | Y | Y | U | Y | N | Y | U | Y | Y | U | Y | N | Y | Y | Y | N | 11 |
| **Timothy 2015** | Y | N | N | Y | Y | Y | U | Y | N | Y | U | Y | Y | Y | Y | N | Y | Y | Y | Y | 14 |
| **Vanessa 2012** | Y | N | N | U | Y | Y | U | Y | N | Y | U | Y | Y | Y | Y | N | Y | Y | Y | N | 12 |
| **Verena 2017** | Y | N | N | U | Y | Y | U | Y | N | Y | U | Y | Y | Y | Y | N | Y | Y | Y | Y | 13 |
| **Wang 1987** | Y | U | U | U | Y | Y | U | Y | N | Y | U | Y | Y | Y | Y | N | Y | Y | Y | Y | 13 |
| **Wang 2015** | Y | N | N | U | Y | Y | U | Y | N | Y | U | Y | Y | U | Y | N | Y | Y | Y | Y | 12 |
| **Winnie 2018** | Y | N | N | U | Y | Y | U | Y | N | Y | U | Y | Y | Y | Y | N | Y | Y | Y | Y | 13 |
| **Wolfsberger 2003** | Y | N | N | U | Y | Y | U | Y | N | Y | U | Y | Y | U | Y | N | Y | Y | Y | N | 11 |
| **Xin 2011** | Y | N | N | Y | Y | Y | U | Y | N | Y | U | Y | Y | Y | Y | N | Y | Y | Y | Y | 14 |
| **Yan 2015** | Y | N | N | U | Y | Y | U | Y | N | Y | U | Y | Y | Y | Y | N | Y | Y | Y | Y | 13 |
| **Yang 2015** | Y | N | N | U | Y | Y | U | Y | N | Y | U | Y | Y | Y | Y | N | Y | Y | Y | Y | 13 |
| **Yan-Long 2018** | Y | N | N | U | Y | Y | U | Y | N | Y | U | Y | Y | Y | Y | N | Y | Y | Y | N | 12 |
| **Yi 2018** | Y | N | N | U | Y | Y | U | Y | N | Y | U | Y | Y | Y | Y | N | Y | Y | Y | N | 12 |
| **Yi-Jun 2017** | Y | N | N | U | Y | Y | U | Y | N | Y | U | Y | Y | Y | Y | N | N | Y | Y | N | 11 |
| **Youichi 1986** | Y | U | N | U | Y | Y | U | Y | N | Y | U | Y | Y | U | U | N | Y | Y | Y | N | 10 |
| **Youngki 2014** | Y | N | N | U | Y | Y | U | Y | N | Y | U | Y | Y | Y | Y | N | Y | Y | Y | Y | 13 |

Y: Yes; U: Unclear; N: No;

Q1: Was the hypothesis/aim/objective of the study clearly stated?

Q2: Was the study conducted prospectively?

Q3: Were the cases collected in more than one center?

Q4: Were patients recruited consecutively?

Q5: Were the characteristics of the patients included in the study described?

Q6: Were the eligibility criteria for entry into the study clearly stated?

Q7: Did patients enter the study at a similar point in the disease?

Q8: Was the intervention of interest clearly described?

Q9: Were additional interventions (cointerventions) clearly described?

Q10: Were relevant outcome measures established a priori?

Q11: Were outcome assessors blinded to the intervention that patients received?

Q12: Were the relevant outcomes measured using appropriate objective/subjective methods?

Q13: Were the relevant outcome measures made before and after the intervention?

Q14: Were the statistical tests used to assess the relevant outcomes appropriate?

Q15: Was follow-up long enough for important events and outcomes to occur?

Q16: Were losses to follow-up reported?

Q17: Did the study provided estimates of random variability in the data analysis of relevant outcomes?

Q18: Were the adverse events reported?

Q19: Were the conclusions of the study supported by the results?

Q20: Were both competing interests and sources of support for the study reported?
