# Supplementary material for: Are dopamine agonists still the first-choice treatment for prolactinoma in the era of endoscopy? A systematic review and meta-analysis
Source: Chin Neurosurg J. 2022 Apr 8;8:9. doi: 10.1186/s41016-022-00277-1 (PMC8994364; doi:10.1186/s41016-022-00277-1)
Supplement: Supplementary file 14 — Additional file 14: Supplementary file 1. Literature research strategy. [file 41016_2022_277_MOESM14_ESM.docx]

Literature research stratege:

("Prolactinoma/drug therapy"[Mesh] OR "Prolactinoma/surgery"[Mesh] OR "Prolactinoma/therapy"[Mesh] OR prolactinomas OR (lactotroph adenoma) OR (adenoma, lactotroph) OR (adenomas, lactotroph) OR (lactotroph adenomas) OR (prolactinoma, familial) OR (prl-secreting pituitary adenoma) OR (prl secreting pituitary adenoma) OR (prl-secreting pituitary adenomas) OR (pituitary adenoma, prl-secreting) OR (pituitary adenomas, prl-secreting) OR (prolactin-producing pituitary adenoma) OR (pituitary adenoma, prolactin-producing) OR (pituitary adenomas, prolactin-producing) OR (prolactin producing pituitary adenoma) OR (prolactin-producing pituitary adenomas) OR (prolactin-secreting pituitary adenoma) OR (prolactin secreting pituitary adenoma) OR (adenoma, prolactin-secreting, pituitary) OR (pituitary adenoma, prolactin-secreting) OR (pituitary adenoma, prolactin secreting) OR (pituitary adenomas, prolactin-secreting) OR prolactin-secreting pituitary adenomas OR microprolactinoma OR microprolactinomas OR macroprolactinoma OR macroprolactinomas OR Prolactinoma* OR lactotroph Adenoma* OR prl-secreting pituitary Adenoma* OR prl-secreting pituitary Adenoma* OR prolactin-producing pituitary adenoma OR Microprolactinoma* OR Macroprolactinoma* OR (giant prolactinoma)) AND ("Dopamine Agonists"[Mesh] OR (agonists, dopamine) OR (receptor agonists, dopamine) OR (dopamine receptor agonist) OR (agonist, dopamine receptor) OR (receptor agonist, dopamine) OR (dopamine receptor agonists) OR (dopaminergic agonists) OR (agonists, dopaminergic) OR (dopaminergic agonist) OR (agonist, dopaminergic) OR (agonists, dopamine receptor) OR (dopamine agonist) OR (agonist, dopamine) OR "Endoscopy"[Mesh] OR (Surgical Procedures, Endoscopic) OR (Procedure, Endoscopic Surgical) OR (Procedures, Endoscopic Surgical) OR (Surgical Procedure, Endoscopic) OR (Endoscopy, Surgical) OR (Surgical Endoscopy) OR (Endoscopic Surgical Procedure) OR Endoscopic Surgical Procedures OR "Microscopy"[Mesh] OR (optical microscopy) OR (microscopy, optical) OR (simple microscopy) OR (microscopy, simple) OR (light microscopy) OR (microscopy, light) OR (compound microscopy) OR (microscopy, compound) OR (hand-held microscopy) OR (hand held microscopy) OR (microscopy, hand-held) OR dopamine agonist* OR bromocriptine OR cabergoline)
