# Supplementary material for: Are dopamine agonists still the first-choice treatment for prolactinoma in the era of endoscopy? A systematic review and meta-analysis
Source: Chin Neurosurg J. 2022 Apr 8;8:9. doi: 10.1186/s41016-022-00277-1 (PMC8994364; doi:10.1186/s41016-022-00277-1)
Supplement: Supplementary file 2 — Additional file 2: Supplementary Figure 2. Forest plots for subgroup analysis of biochemical cure rates in surgery-treated patients subgrouped by patients type (A), publication years (B), surgery types (C); and in DAs-treated patients subgrouped by patients type (D), publication years (E), DAs types (F). [file 41016_2022_277_MOESM2_ESM.pdf]

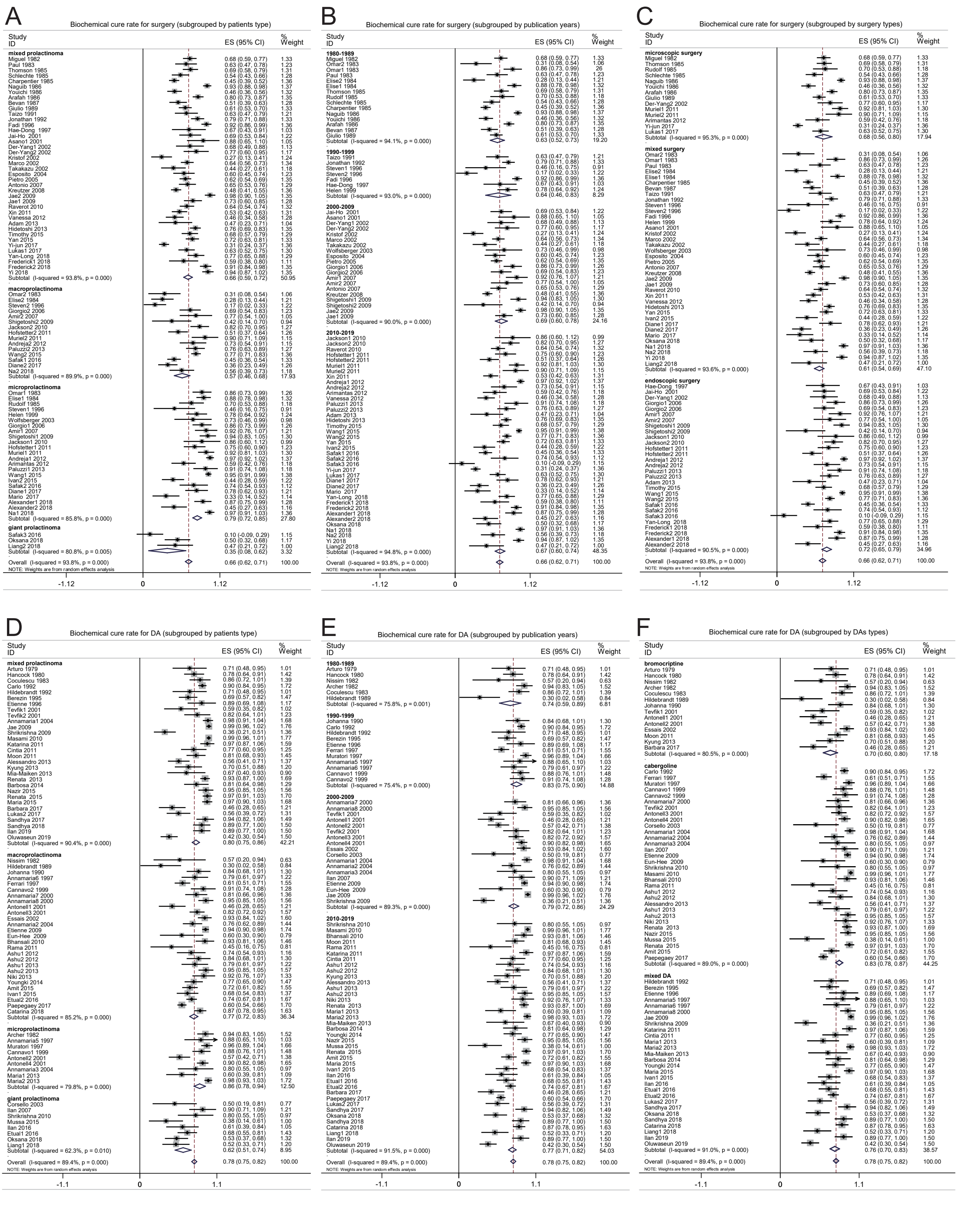
A
Biochemical cure rate for surgery (subgrouped by patients type)
Study ID
ES (95% CI)
% Weight
mixed prolactinoma
Subtotal (I-squared = 93.8%, p = 0.000) 0.66 (0.59, 0.72) 50.95
macroprolactinoma
Subtotal (I-squared = 89.9%, p = 0.000) 0.57 (0.46, 0.68) 17.93
microprolactinoma
Subtotal (I-squared = 85.8%, p = 0.000) 0.79 (0.72, 0.85) 27.80
giant prolactinoma
Subtotal (I-squared = 80.8%, p = 0.005) 0.35 (0.08, 0.62) 3.32
Overall (I-squared = 93.8%, p = 0.000) 0.66 (0.62, 0.71) 100.00
NOTE: Weights are from random effects analysis
-1.12
0
1.12
B
Biochemical cure rate for surgery (subgrouped by publication years)
Study ID
ES (95% CI)
% Weight
1980-1989
Subtotal (I-squared = 94.1%, p = 0.000) 0.63 (0.52, 0.73) 19.20
1990-1999
Subtotal (I-squared = 93.0%, p = 0.000) 0.64 (0.46, 0.83) 8.29
2000-2009
Subtotal (I-squared = 90.0%, p = 0.000) 0.69 (0.60, 0.78) 24.16
2010-2019
Subtotal (I-squared = 94.8%, p = 0.000) 0.67 (0.60, 0.74) 48.35
Overall (I-squared = 93.8%, p = 0.000) 0.66 (0.62, 0.71) 100.00
NOTE: Weights are from random effects analysis
-1.12
0
1.12
C
Biochemical cure rate for surgery (subgrouped by surgery types)
Study ID
ES (95% CI)
% Weight
microscopic surgery
Subtotal (I-squared = 95.3%, p = 0.000) 0.68 (0.56, 0.80) 17.94
mixed surgery
Subtotal (I-squared = 93.6%, p = 0.000) 0.61 (0.54, 0.69) 47.10
endoscopic surgery
Subtotal (I-squared = 90.5%, p = 0.000) 0.72 (0.65, 0.79) 34.96
Overall (I-squared = 93.8%, p = 0.000) 0.66 (0.62, 0.71) 100.00
NOTE: Weights are from random effects analysis
-1.12
0
1.12
D
Biochemical cure rate for DA (subgrouped by patients type)
Study ID
ES (95% CI)
% Weight
mixed prolactinoma
Subtotal (I-squared = 90.4%, p = 0.000) 0.80 (0.75, 0.86) 42.21
macroprolactinoma
Subtotal (I-squared = 85.2%, p = 0.000) 0.77 (0.72, 0.83) 36.34
microprolactinoma
Subtotal (I-squared = 79.8%, p = 0.000) 0.86 (0.78, 0.94) 12.50
giant prolactinoma
Subtotal (I-squared = 62.3%, p = 0.010) 0.62 (0.51, 0.74) 8.95
Overall (I-squared = 89.4%, p = 0.000) 0.78 (0.75, 0.82) 100.00
NOTE: Weights are from random effects analysis
-1.1
0
1.1
E
Biochemical cure rate for DA (subgrouped by publication years)
Study ID
ES (95% CI)
% Weight
1980-1989
Subtotal (I-squared = 75.8%, p = 0.001) 0.74 (0.59, 0.89) 6.81
1990-1999
Subtotal (I-squared = 75.4%, p = 0.000) 0.83 (0.75, 0.90) 14.88
2000-2009
Subtotal (I-squared = 89.3%, p = 0.000) 0.79 (0.72, 0.86) 24.29
2010-2019
Subtotal (I-squared = 91.5%, p = 0.000) 0.77 (0.71, 0.82) 54.03
Overall (I-squared = 89.4%, p = 0.000) 0.78 (0.75, 0.82) 100.00
NOTE: Weights are from random effects analysis
-1.1
0
1.1
F
Biochemical cure rate for DA (subgrouped by DAs types)
Study ID
ES (95% CI)
% Weight
bromocriptine
Subtotal (I-squared = 80.5%, p = 0.000) 0.70 (0.60, 0.80) 17.18
cabergoline
Subtotal (I-squared = 89.0%, p = 0.000) 0.83 (0.78, 0.87) 44.25
mixed DA
Subtotal (I-squared = 91.0%, p = 0.000) 0.76 (0.70, 0.83) 38.57
Overall (I-squared = 89.4%, p = 0.000) 0.78 (0.75, 0.82) 100.00
NOTE: Weights are from random effects analysis
-1.1
0
1.1
